# Low level genome mistranslations deregulate the transcriptome and translatome and generate proteotoxic stress in yeast

**DOI:** 10.1186/1741-7007-10-55

**Published:** 2012-06-20

**Authors:** João A Paredes, Laura Carreto, João Simões, Ana R Bezerra, Ana C Gomes, Rodrigo Santamaria, Misha Kapushesky, Gabriela R Moura, Manuel AS Santos

**Affiliations:** 1 RNA Biology Laboratory, Department of Biology and CESAM, University of Aveiro, 3810-193 Aveiro, Portugal; 2 European Bioinformatics Institute, Wellcome Trust Genome Campus, Hinxton, CB10 1SD Cambridge, UK

**Keywords:** Yeast, mistranslation, tRNA, protein synthesis, mRNA profiling, stress, proteotoxic stress, protein misfolding, unfolded protein response

## Abstract

**Background:**

Organisms use highly accurate molecular processes to transcribe their genes and a variety of mRNA quality control and ribosome proofreading mechanisms to maintain intact the fidelity of genetic information flow. Despite this, low level gene translational errors induced by mutations and environmental factors cause neurodegeneration and premature death in mice and mitochondrial disorders in humans. Paradoxically, such errors can generate advantageous phenotypic diversity in fungi and bacteria through poorly understood molecular processes.

**Results:**

In order to clarify the biological relevance of gene translational errors we have engineered codon misreading in yeast and used profiling of total and polysome-associated mRNAs, molecular and biochemical tools to characterize the recombinant cells. We demonstrate here that gene translational errors, which have negligible impact on yeast growth rate down-regulate protein synthesis, activate the unfolded protein response and environmental stress response pathways, and down-regulate chaperones linked to ribosomes.

**Conclusions:**

We provide the first global view of transcriptional and post-transcriptional responses to global gene translational errors and we postulate that they cause gradual cell degeneration through synergistic effects of overloading protein quality control systems and deregulation of protein synthesis, but generate adaptive phenotypes in unicellular organisms through activation of stress cross-protection. We conclude that these genome wide gene translational infidelities can be degenerative or adaptive depending on cellular context and physiological condition.

## Background

Genome decoding fidelity is essential to maintain cell homeostasis and fitness in all organisms. However, errors in DNA transcription, pre-mRNA splicing and editing, and in mRNA translation, generate mutant proteins whose toxicity creates homeostatic imbalances (proteotoxic stress). At the gene translation level, missense, nonsense, frameshifting and ribosome drop-off errors affect protein synthesis [1]. Missense errors arise from incorrect tRNA selection by the ribosome or incorrect tRNA aminoacylation by aminoacyl-tRNA synthetases (aaRSs) and occur with average frequency of 10^-3 ^to 10^-5 ^per codon decoded [2-4]. Such errors are codon-dependent and are sensitive to the nutritional status of the cell [5,6]. Translational frameshifting errors occur at a frequency of 10^-5 ^and are caused by tRNA slippage during mRNA decoding [1], while read-through of stop codons (nonsense errors) results from competition between non-sense suppressor tRNAs and release factors (RFs) and occur at a frequency of 10^-3 ^[7]. Ribosome drop-off errors are poorly understood but have a basal frequency of 4 × 10^-4 ^at ribosome pausing sites [8,9].

Eukaryotic cells mitigate the deleterious effects of those gene expression infidelities through the ubiquitin-proteasome pathway (UPS), autophagy, ER-associated protein degradation pathway (ERAD) and molecular chaperones [10,11]. Despite this, mutations that affect protein synthesis efficiency and/or accuracy cause neurodegenerative disease in mice and various human diseases, including mitochondrial diseases and cancer [reviewed in [12]]. For example, a single mutation in the editing domain of the mouse alanyl-tRNA synthetase (AlaRS) leads to serine (Ser) misincorporation at alanine (Ala) codons and causes rapid loss of Purkinje cells [13], while mischarging of the tRNA^Met ^with homocysteine (Hcy) causes proteome N-homocysteinylation in vascular endothelial cells (HUVEC) and increases the risk of vascular disease in humans [14]. Moreover, reactive oxygen species (ROS) modify phenylalanine (Phe) to m-tyrosine (m-Tyr), o-tyrosine (o-Tyr) and p-tyrosine (Tyr) and promote m-Tyr misincorporation into proteins by both the cytoplasmic and mitochondrial phenylalanyl-tRNA synthetases (PheRS) via mischarging of tRNA^Phe ^(m-Tyr-tRNA^Phe^), but the consequences of proteome m-tyrosylation are not known [15]. Similarly, mutations in mitochondrial tDNA genes encoding tRNA^Phe^, tRNA^Leu^, tRNA^Ser^, tRNA^His ^and tRNA^Lys^, which affect the accuracy and/or efficiency of translation, cause myopathy, encephalopathy, lactic acidosis, stroke-like episodes or myoclonic epilepsy with ragged-red fibers (MELAS/MERRF syndromes) [16-18], indicating that mitochondria are particularly sensitive to gene translation fidelity and efficiency.

Most surprisingly, elevated gene translational errors (mistranslations) can trigger expression of advantageous phenotypes in yeast and bacteria [19-22]. For example, misincorporation of Ser at Leu CUG codons allows yeast to grow in the presence of high concentrations of arsenite, cadmium, cycloheximide, NaCl and H_2_O_2 _[20,21], while natural epigenetic control of both stop codon read-through and antizyme frameshifting by the [*PSI^+^*] prion generates phenotypic diversity and regulates the cellular concentration of polyamines [23-25]. In the fungal pathogen *Candida albicans *such mistranslations generate extensive phenotypic diversity, induce expression of novel colony and cell morphotypes and are associated with evolution of a genetic code alteration [26,27].

Mistranslations are also used to synthesise statistical proteins of high potential to generate antigenic variation in *Mycoplasma *species which encode threonyl-, phenylayl- and leucyl-tRNA synthetases (ThrRS, LeuRS and PheRS, respectively) with defective amino acid editing domains [28]. In *E. coli*, mistranslations induce a hypermutagenic phenotype known as translational stress mutagenesis (TSM) [29,30], raising the fascinating hypothesis that phenotypic outcomes of gene translational errors can be rapidly fixed in the genome. We unveil below hidden features of the biology of genome translational infidelities which help us understand some of the phenotypes described above.

## Results

### Model system to study gene mistranslations in a controlled manner

**Figure 1:**
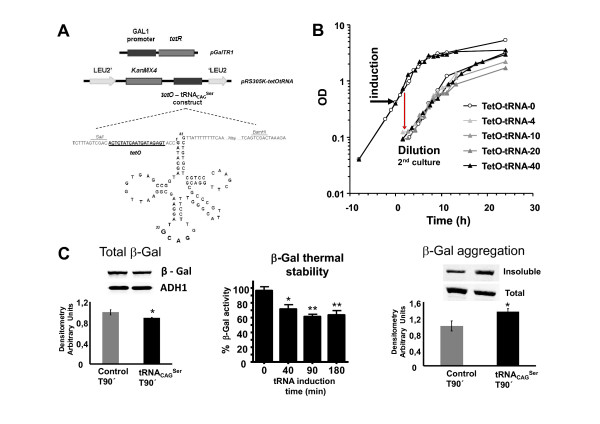
**Engineering regulated expression of a heterologous tRNA_CAG_^Ser ^in yeast**. **A) **The recombinant mistranslating tRNA_CAG_^Ser ^gene (cloned into plasmid pRS305K-*tetO*-tRNA) was integrated into the yeast *LEU2 *locus by homologous recombination using the *KanMX4 *gene as a selectable marker. The same yeast strain was transformed with the *pGalTRI *plasmid containing the *GAL1 *promoter - *tetR *construct. Selection was carried out in MMgalactose-URA containing geneticin (200 mg/L). **B) **Growth curves of *tetO*-tRNA clones growing in liquid MMgalactose+geneticin at 30°C. Expression of the tRNA_CAG_^Ser ^was induced by addition of 40 μg/mL of tetracycline at OD_600 _= 0.4 to 0.5 (T0'). Yeast growth was monitored by measuring OD_600 _of the culture or by counting the number of cells per mL using a Neubauer cell counting chamber. The dilution shown indicates start of second cultures where the tetracycline concentrations tested are indicated in the inset key in μg/mL. **C) **Left panel shows the amount of β-gal protein expressed in Control and mistranslating yeast cells at T90'. Center panel shows the residual activity of β-gal after its thermal inactivation at 47°C for 10 minutes. The activity of the β-gal fraction that remained functional after thermal inactivation and refolding (4°C) was determined by incubating cell extracts at 37°C for two minutes in the presence of ONPG. The values in the graph represent activity in *tetO*-tRNA cells as percent relative to Control cells. The right panel shows increased aggregation of mistranslated β-gal relative to wild type enzyme, confirming that mistranslation is an important source of protein aggregation. The *P*-values for statistical comparisons (two-tailed unpaired Student's *t*-test) between *tetO*-tRNA and Control cells in each graph are shown - *P < 0.05; **P < 0.01.

Gene mistranslations have been studied over the years using the aminoglycosidic antibiotics neomycin, streptomycin, ribostamycin and paromomycin and nonsense or missense suppressor tRNAs [29-35]. These studies helped in the understanding of the mechanisms of antibiotic action and mRNA decoding by the ribosome, but failed to unveil positive and degenerative roles of mistranslations, which are fundamental to fully understanding the biology of gene translational errors. In order to overcome these limitations, we have engineered regulated codon misreading in yeast using a tRNA_CAG_^Ser ^(Figure 1A) that misreads leucine CUG codons as Ser (tRNA_CAG_^Ser^). Since the yeast genome contains 30,994 CUG codons distributed over 88.8% of its genes, the mutant tRNA_CAG_^Ser ^misincorporates Ser on a proteome-wide scale [36-38], providing an ideal system to study global effects of gene mistranslations. In order to regulate these mistranslations, the tRNA_CAG_^Ser ^was expressed under the control of the *E. coli *Tet operator (*tetO*) - Tet repressor protein (*tetR*) system [39]. TetR expression was driven by the yeast *GAL1 *promoter in medium containing galactose (*GAL1 *ON) as the sole carbon source (Figure 1A). Addition of tetracycline to this growth medium inhibits the *tetR *protein allosterically, clears *tetO *and activates transcription of the mutant tRNA_CAG_^Ser^.

Since biologically and biomedically relevant gene mistranslations occur at levels that do not compromise cell viability, we have attempted to determine the mistranslations' induction time and intensity thresholds that produced minimal impact on growth rate. Expression of the tRNA_CAG_^Ser ^could be induced with 40 μg/ml of tetracycline at an OD_600 _of 0.4 to 0.5 without significant alteration in growth rate, small differences were visible in stationary phase only (Figure 1B). Earlier induction of the tRNA (OD_600 _= 0.1) resulted in higher reduction of cell density in stationary phase and slowed growth of cells diluted into fresh medium (Additional file 1, Figure S1). Putting it simply, gene mistranslations remained phenotypically silent during the first three to four yeast generations but their negative effects increased in intensity over time, as one would predict from gradual accumulation of the mistranslating tRNA. When cells expressing the tRNA_CAG_^Ser ^were spotted in MMgalactose agar plates, a decrease in viability or ability to re-grow and form colonies was observed. This effect was stronger when a higher concentration of tetracyclin was used (Additional file 2, Figure S2A). A similar result was observed when Control and *tetO*-tRNA cells pre-cultured in MMgalactose were directly plated in MMgalactose + tetracycline agar plates (Additional file 2, Figure S2B), indicating that mistranslations become degenerative overtime.

In order to confirm the misreading activity of the tRNA_CAG_^Ser^, we have co-expressed the *E. coli *β-galactosidase (β-gal) and the tRNA_CAG_^Ser ^genes in the same recombinant cells. The *E. coli LacZ *gene contains 54 CUG codons and misincorporation of Ser at these Leu-codons generates a combinatorial array of mutant β-gal molecules (statistical β-gal) whose altered stability can be quantified using thermal denaturation and aggregation assays [40,41]. The high number of CUG codons present in the *LacZ *gene combined with the different chemical properties of Ser (polar amino acid) and Leu (hydrophobic amino acid) make β-gal a highly sensitive reporter, allowing for monitoring low level misreading activity of the tRNA_CAG_^Ser^. As expected, Ser misincorporation at CUGs decreased the cellular concentration of β-gal (Figure 1C, left panel) and a thermal denaturation assay [36] showed decreased β-gal activity after heat denaturation and refolding (T40' - 25.1% and T90' - 35.0%) (Figure 1C, center panel). Mistranslated β-gal also had higher propensity to aggregate (Figure 1C, right panel), confirming previous data on the role of gene mistranslations on protein aggregation [13]. We have attempted to quantify the expression of the tRNA_CAG_^Ser ^by Northern blot analysis but we were unable to do so. This was consistent, however, with our previous quantitative mass-spectrometry studies which showed that constitutive expression of the tRNA_CAG_^Ser ^in yeast leads to 1.4% misincorporation of Ser at Leu CUG positions, but the tRNA was very difficult to detect by Northern blot analysis [20].

### General features of the transcriptional response to gene mistranslations

**Figure 2:**
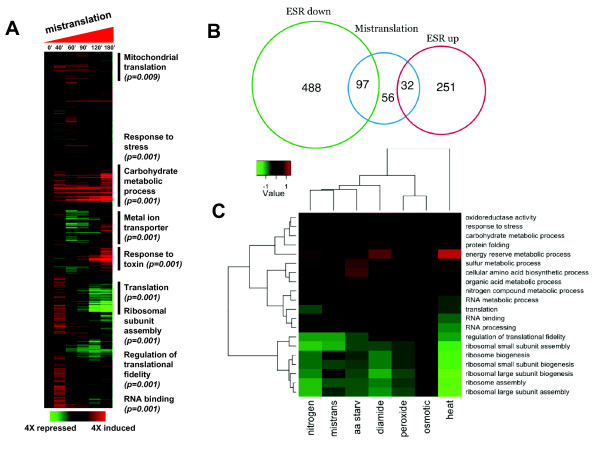
**Transcriptional responses of yeast exposed to gene mistranslations and environmental stressors**. **A) **Gene expression profiles of mistranslating cells at T0', T40', T60', T90', T120' and T180'. **B) **Overlap of genes differentially expressed (DEGs) in the Environmental Stress Response (ESR) and in mistranslations (> 2-fold deregulation). Approximately 70% of the mistranslations DEGs are related to the stress response. The overlap of genes up-regulated by the ESR and mistranslations increased over time reaching 82% at mistranslation T180'. Similarly, the overlap of down-regulated genes increases significantly at mistranslations T120' and T180'. **C) **Summary of GO terms of ESR and mistranslations DEGs. Each color square represents the average expression level of the genes annotated with the corresponding GO term for each stress condition. Stress conditions have been hierarchically clustered. Mistranslations activate stress responders and repress translational and ribosomal biogenesis processes, although average fold variation is not as strong as for heat shock or nitrogen depletion. The ESR up- and down-regulated gene lists (*ESRup*, *ESRdown*) were obtained from Gasch *et al. *[42].

The transcriptional response to gene mistranslations was investigated by profiling the transcriptome of yeast cells at the mistranslations time points of T0', T40', T60', T90', T120' and T180'. The global gene expression deregulation pattern (Figure 2A) was consistent with induction of the yeast environmental stress response (ESR) [42]. Mistranslating cells shared 97 down-regulated and 32 up-regulated genes (> 2-fold deregulation) with the Control cells exposed to environmental stressors and deregulated 56 genes, which were not found in the ESR gene list [42] (Figure 2B; Additional file 3, Figure S3; Additional file 4, Table S2; Additional file 5, Table S3), after moderated t-tests with *P*-value cut-off 0.05 after multiple testing correction. Gene enrichment analysis using gene ontology (GO) terms confirmed that genes belonging to oxidative and general stress, carbohydrate and energy reserve metabolism, protein folding and sulphur metabolism were up-regulated, while genes encoding translational factors, ribosome biogenesis and assembly were down-regulated (Figure 2A, C; Table 1; Additional file 3, Figure S3; Additional file 6, Figure S4). Genes encoding ribosomal proteins were weakly down-regulated up to T90', but a strong down-regulation effect was observed at T180'. In contrast, up-regulation of molecular chaperones and trehalose biosynthesis genes was clearly visible at T40' (Figure 2A; Additional file 6, Figure S4; Additional file 7, Table S3). The initial response (T40') to gene mistranslations also involved up-regulation of translation and metabolic processes, but their deregulation changed from positive to negative over time (Figure 2A). A cross comparison of deregulated genes (DEGs) using GO terms enrichment confirmed the negative effect of mistranslations on the protein synthesis machinery and highlighted important similarities with the ESR (Figure 2C). During the initial stages of mistranslations (T0' to T90') DEGs shared with the ESR were essentially up-regulated, while at T120' and T180' common up- and down-regulated DEGs were detected (Additional file 5, Table S2). This indicates that molecular chaperones and other stress genes were the first line of defense against the gene mistranslations while down-regulation of protein synthesis genes happened later (Figure 2A).

**Table 1:** General features of the transcriptional response to genome mistranslations

GO term	Genes in term	T40' genes	*P*-value	T60' genes	*P*-value	T90' genes	*P*-value	T120' genes	*P*-value	T180' genes	*P*-value
**protein targeting to mitochondrion**	53	18	3·10^-10^								

**regulation of protein metabolic process**	276	48	2·10^-12^			35	3·10^-10^			51	2·10^-25^

**transposition**	135			32	3·10^-22^	40	2·10^-25^	28	2·10^-20^		

**macromolecule biosynthetic process**	1675	106	4·10^-10^			106	3·10^-9^			133	8·10^-32^

**vacuolar protein catabolic process**	118			15	5·10^-7^	15	4·10^-5^	15	2·10^-8^	14	3·10^-5^

**response to heat**	198	21	5·10^-3^	16	8·10^-5^	19	2·10^-4^	19	3·10^-8^	17	3·10^-4^

**response to toxin**	31			5	1·10^-3^			5	5·10^-4^	8	5·10^-6^

**translation**	731	124	3·10^-32^			102	2·10^-35^	32	5·10^-5^	131	4·10^-75^

**regulation of translation**	190	47	2·10^-18^					11	3·10^-3^	50	1·10^-32^

**regulation of translational fidelity**	15					5	2·10^-4^	3	3·10^-3^	7	2·10^-7^

**maturation of SSU-rRNA**	94					20	3·10^-10^	8	7·10^-4^	26	1·10^-17^

**ribosome assembly**	69	13	1·10^-4^			16	4·10^-9^			24	5·10^-19^

**ribosome biogenesis**	360	33	6·10^-3^			43	1·10^-11^	16	4·10^-3^	60	1·10^-27^

For each of the time-point DEGs, a hypergeometric test was applied over each GO biological process (R package *GOstats*), selecting GO terms with *P*-value lower than 10^-3^. The GO terms considered for mistranslations are either enriched for four or more time points, or have a significance level below a *P*-value of 10^-9 ^for a single time point. This method provides about 40 GO terms that, after manually removing redundant and generic terms, result into the terms displayed in Table 1.

**Figure 3:**
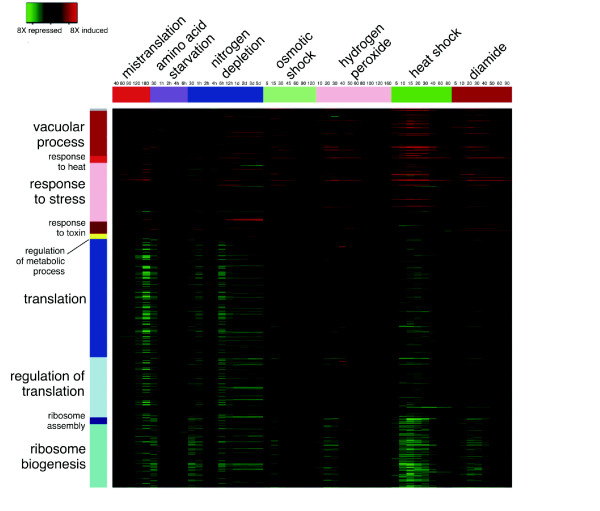
**High overlap of genes deregulated by mistranslations and environmental stressors**. Genes whose expression was deregulated (DEGs) by mistranslations were selected and their expression pattern was compared across environmental stress time points. GO terms enrichment analysis showed the main functional categories affected by mistranslations and environmental stressors. Condition time-points are indicated as small numbers below the stress type (time units are minutes unless otherwise stated: h, hours, d, days). Transcriptional data of yeast responses to environmental stress were obtained from Gasch *et al. *[42].

A cross stress analysis of DEGs further supported the similarities between the stress responses induced by mistranslations and environmental stressors (Figure 3; Table 1). GO terms enrichment analysis showed that genes involved in vacuolar catabolic processes, heat and general stress, response to toxin, regulation of metabolic processes and vacuolar processes were up-regulated (Figure 3; Table 1). Mistranslations had a strong positive impact on the expression of genes encoding small molecular chaperones, namely Hsp26 (8.8-fold), Hsp31 (3.1-fold) and Hsp42 (5.6-fold), which bind aggregated proteins and help in their disaggregation by Hsp70 *SSA3 *(1.5-fold) or *SSA4 *(4.3-fold), Hsp104 (3.5-fold) and Hsp78 (3.1-fold) (Additional file 6, Figure S4; Additional file 7, Table S3; Additional file 8, Figure S5). This supported our observation (Figure 1C) and studies from other laboratories showing that gene mistranslations are an important source of protein misfolding and aggregation [13,43]. Genes encoding proteins involved in metabolic pathways (*ALD3, GND2, SOL4, YDL124W*), synthesis of osmolites, energy reserve and protein stabilization metabolites (*TSL1*), ubiquitin-proteasome pathway (*RNP*s, *UBC*s and *PRE*s), NAD metabolism (*YNL134C, GND2, PNC1*), cell wall remodelling (*YGP1*) and the regulator of the m^7^G-oligoribonucleotide metabolism (*DCS2*), were also up-regulated (Additional file 6, Figure S4; Additional file 7, Table S3). The latter inhibits the hydrolase involved in mRNA decapping (Dcs1) and is regulated by Msn2/4p and the RAS-cAMP-PKA signalling pathway, suggesting that CAP-dependent translation initiation may be strengthened under mistranslations and that part of the transcriptional response to proteotoxic stress is likely modulated by the RAS-PKA signalling pathway [44,45].

The cross stress comparison of the complete set of DEGs corroborated and highlighted the generalized down-regulation of the protein synthesis machinery, in particular of genes encoding translation factors, RNA binding and processing proteins, regulation of translational fidelity, ribosomal proteins and ribosome biogenesis and assembly genes (Figures 2 and 3; Additional file 3, Figure S3; Additional file 6, Figure S4; Additional file 7, Table S3). It also showed down-regulation of chaperones linked to the ribosome (CLIPS network), which fold newly synthesized proteins emerging from it (Additional file 7, Table S3; Additional file 8, Figure S5). These CLIPS included Hsp70s *SSB2*, the Hsp70 partners *SSZ1 *and *ZUO1*, the chaperonin TriC/*CCT*s (*TCP1 *and *CCT2 *- *CCT8*) and members of the prefoldin GimC protein family (*GIM3*, *GIM4 *and *GIM5*), suggesting that down-regulation of the protein synthesis machinery exacerbates protein folding problems caused by gene mistranslations.

### Gene mistranslations affect protein synthesis

**Figure 4:**
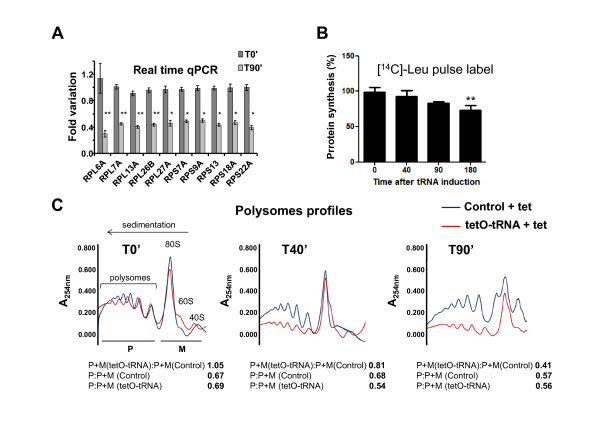
**The effect of gene mistranslations on protein synthesis**. **A) **Validation of expression of ribosomal protein genes by real time quantitative PCR (n = 3) confirmed the down regulation of ribosome biogenesis (*P < 0.05; **P < 0.01 for T90' *vs*. T0' two-tailed unpaired Student's *t*-test comparison). **B) **Mistranslations decreased protein synthesis. Control and mistranslating cells (12 OD_600 _units) were pulse labeled with 62.5 μCi of [^14^C]-Leu for 30' at 30°C. Labeled cells were then disrupted and cleared protein extracts (30 μl) were applied onto paper filters for counting incorporated radioactivity using a scintillation counter. The values in the graph represent incorporated radioactivity in *tetO*-tRNA cells as percent relative to Control cells. The *P*-values for statistical comparisons (two-tailed unpaired Student's *t*-test) between *tetO*-tRNA and Control cells are shown - **P < 0.01. **C) **Polysomal profiles of yeast cells mistranslating at T0', T40' and T90', showing reduction in the number of polysomes engaged in mRNA translation. The relative area of the polysomal (P) *vs*. total area (P+M) of Control and *tetO*-tRNA cells at each time point and the ratios between total polysomes+monosome material (P+M) in *tetO*-tRNA cells and in Control cells before (T0') and after mistranslation induction (T40' and T90'), are shown.

The generalized down-regulation of protein synthesis genes without clear effects on yeast growth rate prompted us to validate the expression of several ribosomal protein genes by RT-qPCR, but the latter confirmed the down-regulation trend detected by the DNA microarrays (Figure 4A; Additional file 7, Table S3; Additional file 9, Figure S6). In order to determine whether the gene expression deregulation had direct impact on protein synthesis, we have also pulse-labelled Control and mistranslating cells with [^14^C]-Leu and analysed their polysome profiles (Figure 4B, C). Reduced protein synthesis and polysome levels were detected (T40' to T90') (Figure 4B, C) and the latter were altered as early as T40', suggesting that down-regulation of protein synthesis accompanied the early up-regulation of the molecular chaperones mentioned above. The T40'and T90' profiles did not show increased levels of monosomes or free ribosomal subunits, indicated by similar P:P+M ratios of Control and *tetO*-tRNA cells at each time point. The reduction of 60% of total P+M material in *tetO*-tRNA cells relative to Control cells at T90' suggests that the subunits released from the polysomes were degraded or may have been incorporated into P-bodies or stress granules. The loss of translational material at T90' (Figure 4C) reduced protein synthesis rate by 15%, as measured by [^14^C]-aa pulse-labeling of proteins (Figure 4B). The higher loss of polysomes (60%) suggested a stronger negative impact on the rate of protein synthesis; however, polysomes content and protein synthesis rate may not be directly correlated due to differences in the methodologies used to quantify both variables. In any case, the lower decrease in protein synthesis rate is consistent with the smaller impact of mistranslation on growth rate (Figure 1B).

**Figure 5:**
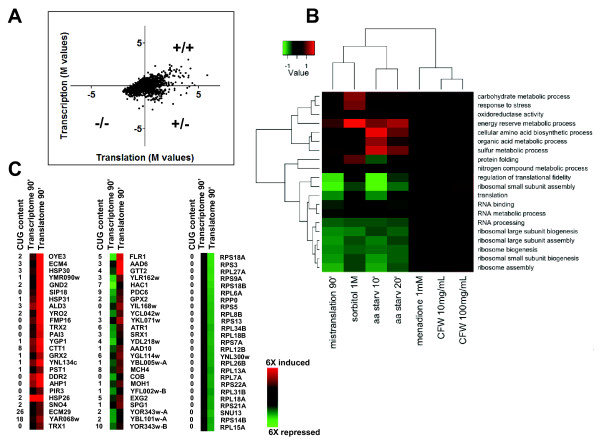
**The translatome of mistranslating cells**. **A) **Graphical representation of the correlation between the profiles of total mRNA (transcriptome) and polysome-associated (translatome) mRNA. The y-axis represents M values of the ratio of total mRNA transcript fractions at time-points T90' and T0'. The x-axis represents M values of the ratio of polysome-associated transcripts at time-points T90' and T0'. **B) **Cross stress comparison of translatome DEGs using GO terms enrichment analysis [99]. Mistranslation translatome DEGs (T90') were used for this comparative analysis. The translatome data of cells exposed to environmental stress were obtained from Halbeisen *et al. *[46]. **C) **Schematic representation of genes shown in the panel-A quadrants, which have higher variation of fold values between the transcriptome and translatome. The data show that gene expression deregulation was independent of the CUG content and that ribosomal proteins are not directly affected by Ser misincorporation at CUGs.

The above observations and the possible increase in mRNA capping activity due to up-regulation of the *DCS2 *gene (see above) led us to analyse the yeast translatome, that is, the fraction of mRNAs that were effectively translated in mistranslating cells. For this, mRNAs were extracted from polysomes and were hybridized onto DNA microarrays as above. A direct comparison of the log2 expression ratios (M values) between the polysomal and total mRNA fractions at mistranslations time T90' showed homo-directional expression variation (both positive and negative) between transcription and translation for most genes (Figure 5A; Additional file 7, Table S3). Analysis of the genes that had fold variation > 1.5 or < -1.5 allowed us to identify 280 genes with similar variation at both transcriptome and translatome levels: 142 genes were up-regulated and 138 genes were down-regulated in both analyses, overlap with likelihood *P *< 0.001 (hypergeometric test) (Figure 5A; Additional file 7, Table S3). A cross stress analysis of the DEGs of the mistranslation translatome (T90'), environmental stress translatome (data obtained from Halbeisen *et al. *[46]) and GO terms enrichment analysis further supported the down-regulation of protein synthesis processes and the up-regulation of the stress response (Figure 5B). More interestingly, it clustered mistranslations at T90' with stronger stressors, namely sorbitol (1M) and amino acid starvation, further confirming that yeast cells integrated the gene mistranslation effects as a strong rather than as a weak stressor [46] (Figure 5B; Additional file 10, Figure S7).

The up-regulated genes are mainly involved in the general and oxidative stress responses (*HSP30*, *SIP18*, *HSP31*, *ALD3*, *TRX2*, *YGP1*, *CTT1*, *GRX2 *and *DDR2*) and unfolded protein binding (*HSP26*, *SNO4*, *HSP31 *and *HSP30*), confirming that some of the transcriptionally up-regulated genes were being translated (Figure 5A, C; Table 2; Additional file 7, Table S3). A similar result was obtained for genes with negative fold variation which were involved in ribosome assembly and translation (Figure 5A, C; Table 2; Additional file 7, Table S3). The list of down-regulated genes included ribosomal protein genes and structural acidic proteins of the ribosome, namely protein P1 alpha (*RPP1A*), protein P2 alpha (*RPP2A*) and protein P0 (*RPP0*), which form a pentameric complex (P0-(P1-P2)_2_) on the ribosomal 60S subunit (ribosome stalk) which stimulates translation factor-dependent GTP hydrolysis [47,48]. The *SNU13 *gene, which encodes a RNA binding protein involved in rRNA processing by the U3 snoRNP and in mRNA splicing through the U4/U6-U5 tri-snoRNP [49,50], was also down-regulated (Figure 5A, C; Additional file 7, Table S3). Interestingly, the *RPS18B *and *RPS18A *genes, which encode structural proteins of the cytosolic (40S) and mitochondrial (30S) ribosome small subunits [51,52], appeared in this restricted list of deregulated genes, suggesting that gene mistranslations had a negative impact on both cytoplasmic and mitochondrial translation.

**Table 2:** GO enrichment for the different groups of genes identified in the transcriptome and translatome comparison

GO term	Genes in GO term	Group	Genes in group	*P*-value	In group and term
**Response to oxidative stress**	83	**++**	142	6.1·10^-9^	14

**Response to stress**	813	**++**	142	7.9·10^-18^	60

**Protein unfolding**	3	**++**	142	0.002	2

**Protein refolding**	17	**++**	142	0.0006	4

**Protein folding**	113	**++**	142	0.0003	10

**Ribosome biogenesis**	355	**--**	138	2.5·10^-67^	83

**Ribosomal large subunit biogenesis**	73	**--**	138	5.3·10^-26^	27

**Ribosomal small subunit biogenesis**	52	**--**	138	1.4·10^-15^	17

**Ribosome assembly**	69	**--**	138	7.7·10^-24^	25

**Ribosomal large subunit assembly**	39	**--**	138	2.8·10^-12^	13

**Ribosomal small subunit assembly**	16	**--**	138	1.2·20^-6^	6

**Ribosome localization**	43	**--**	138	3.5·10^-9^	11

**Translation**	721	**--**	138	4.0·10^-11^	47

**Regulation of translation**	190	**--**	138	3.8·10^-9^	21

**Regulation of translational fidelity**	15	**--**	138	5.0·10^-2^	2

**Response to drug**	120	**+-**	88	0.09	3

**Drug transport**	20	**+-**	88	0.0007	3

**Multidrug transport**	11	**+-**	88	0.0001	3

**Response to toxin**	31	**+-**	88	0.002	3

**Response to chemical stimulus**	457	**+-**	88	0.02	9

**Transposition, RNA mediated**	71	**+-**	88	9.0·10^-9^	9

**Transposition**	74	**+-**	88	1.3·10^-8^	9

Direct positive **++**, direct negative -- and inverse **+-**; GO enrichment for each group was done using the R package *GOstats; *the *P*-values for the processes described in the text are indicated.

A third category of genes (88 genes) had negative transcriptional and positive translational values (Figure 5A, C; Table 2; Additional file 7, Table S3), indicating that they were regulated at the translational level. Most of these genes encode proteins involved in toxin and chemical stimulus responses (*AAD6*, *AAD10*, *GPX2*, *GTT2 *and *SRX1*) and drug transport, for example, *FLR1*, *ATR1*, *PMA2 *and *AQR1 *(Figure 5C; Table 2; Additional file 7, Table S3). A significant number of genes encoding components of yeast transposons, namely *YBL005W-A*, *YFL002W-B*, *YOR343W-A*, *YBL101W-A *and *YOR343W*, appeared in this group (Table 2; Additional file 7, Table S3), suggesting that gene mistranslations generate genome diversity through mobilization of transposon activity.

Unidirectional changes between transcription and translation are associated with a gene expression phenomenon called potentiation [53-55], which is characteristic of specific groups of genes under strong stress intensity [46]. Mistranslations potentiated the expression of the plasma membrane chaperone gene *HSP30 *(16.8-fold), which represses the H(+)-ATPase Pma1, the cell wall protein gene *PST1 *(3.5-fold), which is activated in response to cell wall damage, the oxidative stress genes *GRX2, CTT1, TRX2, ECM4, AHP1, ALD3 *(4.7-, 7.4-, 6.8-, 16.4-, 7.9- and 19.1-fold, respectively), the phospholipid binding protein gene *SIP18 *(11.4-fold), the cell wall secretory glycoprotein gene *YGP1 *(9.2-fold), and the multi-stress protein genes *DDR2 *and *OYE3 *(55.8- and 10.4-fold, respectively). Interestingly, genes that were negatively represented in the total mRNA profile but had positive representation in the translatome profile (T90') (Figure 5A, C; Table 2; Additional file 7, Table S3) were also involved in the stress response. For example, the plasma membrane multidrug transporter gene *FLR1 *(6.2-fold), the phospholipid hydroperoxide glutathione peroxidase gene *GPX2 *(2.9-fold), the bZIP transcription regulator of the UPR (*HAC1*) (1.6-fold), the putative aryl alcohol dehydrogenase genes *AAD6 *and *AAD10 *(14.7- and 2.9-fold, respectively), the sulfiredoxin gene *SRX1 *(2.3-fold) whose protein reduces cysteine-sulfinic acid groups in the peroxiredoxins Tsa1 and Ahp1 and contributes to oxidative stress resistance which was further enhanced by overexpression of the glutathione S-transferase gene *GTT2 *(10.3-fold).

### Gene mistranslations activate the unfolded protein response

**Figure 6:**
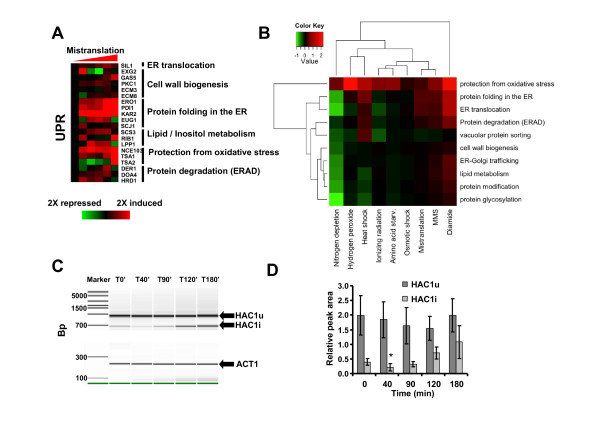
**The UPR is activated by gene mistranslations**. **A) **Gene expression profile highlighting genes involved in the Unfolded Protein Response (UPR) elicited by mistranslations. **B) **Cross stress comparison of DEGs using GO terms enrichment analysis. Mistranslations up-regulate ERAD, ER translocation, protein folding and oxidative stress genes. **C) **Activation of the UPR regulator *HAC1 *(*HAC1i*) through splicing. The panel shows RT-PCR fragments of the *HAC1 *mRNA amplified using specific primers and the presence (*HAC1^u^*) and absence (*HAC1^i^*) of a 252-bp intron in the *HAC1 *primary transcript. A microfluidics gel image showing increased quantity of the *HAC^i ^*isoform at induction times T120' and T180'. **D) **The graph shows the quantification of both forms of the *HAC1 *mRNA displayed in B. The data were normalized to the *ACT1 *internal control (*P < 0.05 for *HAC1*^*i *^*vs*. *HAC1^u ^*two-tailed unpaired Student's *t*-test comparison).

The transcriptome and translatome profiling data strongly suggested that gene mistranslations activated the UPR through the accumulation of misfolded proteins in the ER [56,57]. Indeed, several genes encoding ER resident proteins involved in protein folding and protection from oxidative stress were up-regulated (Figure 6A; Additional file 7, Table S3; Additional file 11, Figure S8). For example, the *KAR2 *gene, which encodes an ATPase with chaperone activity involved in protein import and export into and from the ER and regulates the UPR by interacting with Ire1p [58-60], was up-regulated 1.8-fold at time T90', 2.3-fold at T120' and 2.6-fold at T180' (Figure 6A; Additional file 7, Table S3; Additional file 11, Figure S8). The *SCJ1 *gene whose protein cooperates with Kar2 in protein maturation in the ER, *PDI1 *and *EUG1 *genes which encode proteins involved in disulfide bond formation and unscrambling of non-native disulfide bonds, were all slightly up-regulated by the mistranslations (Figure 6A; Additional file 7, Table S3; Additional file 11, Figure S8). Other genes encoding non-ER resident proteins that are up-regulated by the UPR were also up-regulated. These genes are involved in cell wall remodelling, lipid biosynthesis and in the response to oxidative stress. GO terms enrichment analysis showed that the UPR response to mistranslations is similar to that induced by MMS and affects mainly protein folding, translocation, ERAD and ER oxidative stress (Figure 6B).

Expression of the transcription factor Hac1p, which regulates UPR genes through the UPR enhancer (UPRE) [61-63], was slightly up-regulated at the translatome level at T90' and was down-regulated 6.7-fold at the same time point in the total mRNA profile (Figure 5C; Additional file 7, Table S3). This post-transcriptional regulation of *HAC1 *expression was consistent with processing and activation of the *HAC1 *mRNA since its pre-mRNA contains a 252 bp intron whose retention in the *HAC1 *pre-mRNA renders its mRNA untranslatable (*HAC1^u^*). Splicing of this intron allows for translation of the *HAC1 *mRNA (translatable *HAC1^i^*) and subsequent activation of the UPR via transcription of ER genes [64-68]. The spliced (*HAC^i^*) and unspliced (*HAC1^u^*) forms of *HAC1 *mRNA were detected at T0' by RT-PCR and increased *HAC^i ^*levels were observed between T90' and T180' (Figure 6C, D), confirming that the UPR was activated, explaining the increased transcription of UPR genes containing UPREs from T90' to T180' (Figure 6A). This delay in the activation of the UPR (T90') contrasted with the early detection of mistranslations (T40') (Figure 1C, center panel) and with the early up-regulation of stress-induced chaperones (Additional file 6, Figure S4; Additional file 8, Figure S5). Therefore, steady state activity of proteome quality control systems, in particular of stress-induced molecular chaperones and the ubiquitin-proteasome pathway, likely mitigated the early proteome disruption caused by mistranslations, but above a certain threshold those quality control systems probably became overloaded and proteome quality maintenance required the UPR.

## Discussion

### Regulation of the stress response triggered by mistranslation

The similarities between the transcriptional and translational responses elicited by environmental stressors and the gene mistranslations allow one to get the first insight into the gene regulatory networks involved in the cellular response to genome translational infidelities. Enrichment of transcription factor (TF) binding motifs present in the DEGs promoters (Additional file 12, Figure S9) identified the general stress response element (STRE; AGGGGA/T), the heat-shock responsive element (HSE; nGAAn), the proteasome associated control element (PACE; GGTGGCAAA; targeted by Rpn4p) and the pleiotropic drug resistance element (PDRE; TCCGCGGA targeted by Pdr1p/Pdr3p), as the main *cis *regulatory elements of the transcriptional responses to gene mistranslations (Additional file 12, Figure S9).

The enrichment in STREs (Additional file 12, Figure S9A) indicates that the transcriptional response to gene mistranslations is partly regulated by the cyclic AMP (cAMP) protein kinase A (PKA) (cAMP-PKA) and the TORC1 pathways, which control the transcription factors Msn2p and Msn4p [69,70]. Since ATP and cAMP regulate PKA signalling through the RAS activators (Ras1/2) of the adenylate cyclase Cyr1 [71], gene mistranslations likely decrease cAMP production because Hsp70 Ssa1 regulates positively the guanine nucleotide exchange factor for RAS (Cdc25). Indeed, mistranslated proteins are folding substrates of Hsp70 chaperones and can deviate Ssa1 from its interaction with Cdc25p [72], lowering its activity and decreasing Ras1/2 - Cyr1 activity, cAMP production and PKA activity [73]. The enrichment in STRE elements also provides strong evidence for a role of the TORC1 signalling pathway as it regulates Msn2p/4p by promoting their phosphorylation (see below) [70]. On the other hand, the enrichment in HSE (Additional file 12, Figure S9A) indicates that the observed up-regulation of molecular chaperones is mediated through the heat-shock factor (Hsf1p) [74]. Regulation of Hsf1p involves phosphorylation, conformational alterations and chromatin structure remodelling and it is difficult to understand how gene mistranslations activate it on the sole basis of the comparative transcriptomic studies that we have carried out. Nevertheless, the known down-regulation of Hsf1p via direct interaction with Ssa1-4 members of the Hsp70 family [75] is of particular relevance here as mistranslated proteins likely reduce the pool of free Hsp70 allowing for release and activation of Hsf1p and transcriptional up-regulation of HSE-containing genes.

The enrichment in PACE-containing genes (Additional file 12, Figure S9B) indicates that gene mistranslations up-regulate the UPS through the Rpn4p transcription factor, which is one of the main regulators of proteasome biosynthesis [76,77]. Interestingly, the promoter of the *RPN4 *gene contains HSE (Hsf1p), YRE (Yap1p) and PDRE (Pdr1p/3p) elements and it is likely that mistranslated proteins activate transcription of PACE genes through synergistic interactions between Hsf1p, Yap1p and Pdr1/3p transcription factors. This is consistent with delayed UPS activation under gene mistranslations (Additional file 3, Figure S3; Additional file 7, Table S3; Additional file 13, Figure S10) and suggests that while Hsf1p, Yap1p and Pdr1/3p are directly activated by mistranslated proteins, the UPS is activated by a second wave of transcriptional regulation. An alternative hypothesis is that Rpn4p is stabilized by mistranslated proteins. Rpn4p has a very short half-life under non-stress conditions (approximately two minutes) but is stable under stress [77]; therefore, UPS overloading with mistranslated proteins may stabilize it, providing additional signals for up-regulation of genes encoding proteasome subunits and other PACE genes.

Regarding the up-regulation of stress genes regulated by PDREs (Additional file 12, Figure S9B), there is again an interesting connection with Hsp70 family members as Pdr3p is negatively regulated by Hsp70-Ssa1, while Pdr1p is positively regulated by the CLIP Hsp70 Ssz1 [78,79]. Hence, mistranslated proteins likely activate Pdr3p by freeing it from the repressive interaction with Hsp70-Ssa1/2, suggesting that activation of multidrug response genes is mediated through Pdr3p rather than Pdr1p as the latter is likely down-regulated under mistranslations due to decreased expression of the ribosome-associated activator Ssz1p (Additional file 3, Figure S3; Additional file 8, Figure S5; Additional file 7, Table S3). Mistranslated proteins translocated into mitochondria should also compete for Ssa1/Ssa2 and may activate the retrograde mechanism, which is known to increase multidrug resistance [80]. This is consistent with increased ROS production and deregulation of mitochondrial genes, including the mitochondrial chaperones Hsp78, Hsp60 and Hsp10 by the gene mistranslations.

### The down-regulation of CLIPS, RP and RiBi regulons

Co-down-regulation of CLIPS and the translational machinery is expected to exacerbate the consequences of the gene mistranslations due to the critical role of these chaperones in folding newly synthesized proteins. Indeed, deletion of *SSB1/2 *results in accumulation of misfolded polyubiquitinated proteins and activation of stress HSE regulated genes [81,82], as is also the case in strains harboring deletions in GimC/*GIM *or TriC/*CCT *[83]. The down-regulation of these CLIPS may also explain the high expression of HSPs as the latter are essential for survival in Δ*SSB1/2 *or Δ*GIM*c deleted cells and mildly beneficial in cells lacking the *RAC *complex [82]. Interestingly, accumulation of misfolded proteins is not a major problem in strains lacking *GIM2*, *ZUO1*, *SSZ1 *and *CCT*. Ssb1/2p are the main players in folding newly synthesized proteins while the other CLIPS play alternative roles. Hence, down-regulation of *SSB1/2 *in the mistranslating cells likely increases accumulation of misfolded proteins, which may explain why cells integrated mistranslations as a strong stressor.

## Conclusions

**Figure 7:**
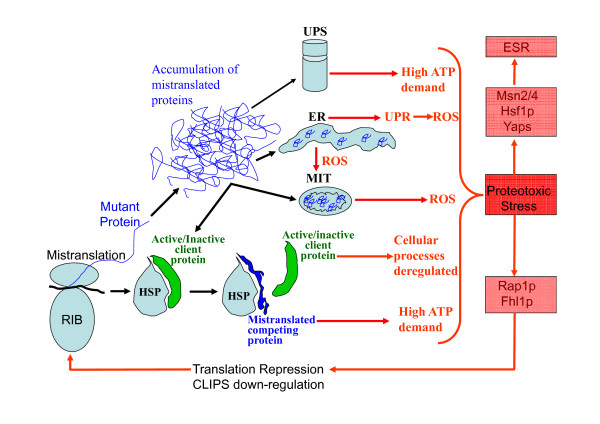
**Working model of the yeast response to gene mistranslations**. Mistranslated proteins are folding substrates of molecular chaperones that compete with wild type client substrates for folding/refolding. Gradual accumulation of mistranslated proteins shifts the HSP-client binding equilibrium to the subpopulation of misfolded proteins, releasing client substrates from HSPs. This activates or inactivates natural HSP-client substrates depending on whether the HSP-client interaction is positive or negative. HSP substrate release deregulates cellular processes mediated by the HSP client proteins and remodels chaperone-chaperone networks, which are critical for cellular homeostasis. Increased protein folding/refolding and degradation increase ATP consumption leading to up-regulation of mitochondrial metabolism and ROS accumulation. Accumulation of mistranslated proteins in mitochondria also increases mitochondrial stress and ROS production. Mistranslated proteins that enter the secretory pathway accumulate in the ER and up-regulate ER resident chaperones, activating the UPR, further increasing ROS production. This leads to a deficit in protein secretion with consequences for cell membranes and cell wall structure and function. Aggregation of mistranslated proteins exacerbates proteotoxic stress, increases ATP consumption and activates the general stress response (ESR) through the transcription factors Msn2/4p and Yaps. Mistranslations also down-regulate ribosome biosynthesis, translational factors and CLIPS, exacerbating the negative consequences of mistranslations due to the critical role of CLIPS in folding newly synthesized proteins.

Our study provides new insight on how genome wide gene mistranslations induce stress resistance and creates phenotypic variability. Activation of the stress response induces a stress cross-protection condition that allows for tolerance to lethal doses of a wide range of environmental stressors [20,21,84]. Stress tolerance in mistranslating cells is, therefore, a secondary effect of ESR activation (Figure 7). The impact of mistranslated proteins on molecular chaperones and on their interaction networks explains the phenotypic diversity generated through gene mistranslations. Indeed, mistranslated proteins are folding substrates of HSPs and CLIPS and their continuous synthesis and accumulation in the cell likely creates chaperones functional insufficiencies that remodel their interaction networks by deviating client substrates (Figure 7; Additional file 14, Figure S11; Additional file 15, Figures S1-S11 legends). Hsp90 illustrates nicely the phenotypic consequences of chaperone overloading. This chaperone is highly interconnected (Additional file 14, Figure S11), assists folding of approximately 1,232 client yeast proteins (approximately 20% of the yeast proteome), in particular of proteins involved in signal transduction and protein trafficking. In mammalian cells, it is also involved in receptor maturation and in innate and adaptive immunity [85]. Disruption of Hsp90 interconnectivity through gene deletions, chemical inhibition or functional overloading, resulted in extensive phenotypic variation (including drug resistance) in yeast, fungi, *Drosophila melanogaster *and *Arabidopsis thaliana *[86-89]. The high interconnectivity of most of the 63 or so yeast chaperones (Additional file 14, Figure S11) suggests, therefore, that mistranslated proteins have high potential to remodel interactions and functions of most chaperones, highlighting avenues to understand the phenotypes associated to gene translational infidelities using systems biology approaches.

## Methods

### Strains construction and growth

The *Saccharomyces cerevisiae *BMA64 strain (EUROSCARF acc. no. 20000D; genotype: MATa/MATα; ura3-52/ura3-52; trp1Δ2/trp1Δ2; leu2-3_112/leu2-3_112; his3-11/his3-11; ade2-1/ade2-1; can1-100/can1-100) was used for the genetic manipulations described below. BMA64 cells were transformed with *pGalTR1 *(kind gift of T. Winckler and T. Dingermann), which encodes the prokaryotic tet-repressor protein (*tetR*) whose expression is activated in the presence of galactose [39]. Yeast transformations were carried out using the lithium acetate method [90]. Clones were grown in MMgalactose-URA (Minimal Medium without uracil: 0.67% yeast nitrogen base, 2% galactose, 0.2% Drop-out mix). For construction of the inducible system, the misreading tRNA_CAG_^Ser ^gene was amplified by PCR and *SalI*/*BamHI *restriction sites were inserted at the 5'- and 3'-ends during the amplification. The tet-operator sequence (*tetO*) was inserted three nucleotides upstream of the mature tRNA 5'-end. These amplified fragments were cloned into the *pRS305K *plasmid [91] yielding the plasmid *pRS305K-tetOtRNA*. These recombinant tRNA genes were integrated into the genome of the yeast strain BMA64A (previously transformed with the plasmid *pGalTR1*) by homologous recombination using linear DNA fragments containing long tails with homology to the *leu2 *integration locus and the geneticin-resistance *KanMX4 *gene. Transformed clones were selected in MMgalactose-URA containing 200 mg/L of geneticin. The integration into the yeast *leu2 *locus were checked by colony PCR followed by Sanger DNA sequencing. For monitoring Ser misincorporation at Leu CUG codons using the β-gal thermal stability assay the above clones were transformed with the pGL-C1 plasmid [36], which encodes a GST-β-gal chimeric gene fusion.

Pre-cultures of yeast cells containing the *tetO*-tRNA cassette (*tetO*-tRNA cells) or the empty cassette (Control cells) were prepared in MMgalactose-URA+geneticin (200 mg/L) media for approximately 16 to 20 hours, at 30°C. Such pre-cultures were used to inoculate fresh cultures of MMgalactose+geneticin (200 mg/L) at OD_600 _of approximately 0.05, which were allowed to grow at 30°C. Tetracycline (40 μg/mL) was then added at OD_600 _0.4 to 0.5. Control cells and *tetO*-tRNA cells were harvested (50 mL) at T0', T40', T60', T90', T120' and T180' of tRNA_CAG_^Ser ^expression induction with tetracycline. Cell pellets were immediately frozen in liquid nitrogen and were stored at -80°C for later use.

### β-galactosidase activity assays

A total of 500 μl of exponentially growing (OD approximately 0.5) Control cells and cells expressing the Ser tRNA_CAG_^Ser ^were harvested at time points T0', T40', T90', T180' after mistranslations induction with tetracycline. Cells were washed and resuspended in 800 μl of Z-buffer (60 mM Na_2_HPO_4_, 40 mM NaH_2_PO_4_·2H_2_O, 10 mM KCl, 1 mM MgSO_4_·7H_2_O, 50 mM 2-mercaptoethanol, pH 7.0), 20 μl of 0.1% SDS and 50 μl of chloroform. Cell suspensions were mixed (vortex) for 30 seconds and incubated in triplicate at 47°C in a water bath for 10 minutes. This β-gal unfolding step was followed by a refolding step, which was carried out by incubating samples on ice for 30 minutes. Residual β-gal activity was then quantified at 37°C. For this, the assay tubes (200 μl) were incubated for five minutes at 37°C and then 4 mg/mL of the *o*-nitrophenyl-β-D-galactopyranoside (ONPG) substrate were added to each tube and reactions were allowed to proceed for two minutes and were stopped by the addition of 400 μl of 1M Na_2_CO_3_. β-gal activity was determined by monitoring *o*-nitrophenol synthesis at 420 nm.

### β-galactosidase aggregation assay

Protein aggregation assays were adapted from [92]. Briefly, 10 A_600 _units of exponentially growing cells were harvested by centrifugation, washed and resuspended in 300 μl of lysis buffer (50 mM potassium phosphate buffer pH 7, 1 mM ethylenediaminetetraacetic acid (EDTA), 5% v/v glycerol, 1 mM phenylmethylsulfonyl fluoride, and complete mini protease inhibitor cocktail from Roche Diagnostics (Mannheim, Germany). Cells were disrupted by vortexing with glass beads (0.5 mm diameter) for 3 × 1 minute, with 1-minute incubation on ice between each disruption cycle. Intact cells were removed by centrifugation of the crude extract at 5,000 rpm for 15 minutes. Aggregated proteins were isolated by centrifugation at 13,000 rpm for 20 minutes and membrane proteins were removed by washing the pellet with a lysis buffer containing 2% Triton X-100. The final pellet was resuspended in 100 μl of lysis buffer.

### Western blot analysis

Total and aggregated protein fractions were analyzed under reducing conditions using 12% SDS-PAGE and blotted onto nitrocellulose membranes according to standard procedures. β-Gal was detected using a rabbit anti-β-Gal primary antibody (Molecular Probes, Leiden, The Netherlands) at 1:5,000 dilution. Bound antibody was visualized by incubating membranes with a IRDye680 goat anti-rabbit secondary antibody (Li-cor Biosciences, Lincoln, NE, USA) at 1:10,000 dilution. Detection was carried out using an Odyssey Infrared Imaging system (Li-cor Biosciences). The amount of aggregated β-Gal was normalized to the amount of β-Gal present in the total protein fraction.

### RNA isolation and labeling

RNA isolation and labeling were carried out as described by van de Peppel [93], with minor modifications. Briefly, total yeast RNA extracts were prepared using hot phenol (T0' to T180'). cDNA synthesis was carried out using 40 μg of total RNA extracted from T0' to T180' samples and SuperscriptII Reverse Transcriptase (Invitrogen, Carlsbad, CA, USA). A pool of mRNAs extracted from Control cells at several time points was used as reference RNA sample. For labeling, all cDNAs were synthesized in presence of aminoallyl-dUTP (Sigma-Aldrich, Munich, Germany), purified using Microcon-30 (Millipore, Billerica, MA, USA) columns and were coupled to Cy3 or Cy5 fluorophores (Amersham Biosciences, Piscataway, NJ, USA). Before hybridization, free dyes were removed using Chromaspin-30 (Clontech, Palo Alto, CA, USA) columns and the efficiency of cDNA synthesis and dye incorporation was measured using a Nanodrop spectrophotometer by determining the full spectrum of absorption in the 190 to 750 nm range and registering the OD values at 260 nm, 550 nm and 649 nm points for each sample. For each hybridization 300 ng of Cy3- and Cy5-labelled cDNAs were mixed with in house printed yeast arrays (YAUAv 1.0, DNA Microarray Facility, Department of Biology, University of Aveiro, Aveiro, Portugal) and hybridized for 20 hours at 42°C using an Agilent (Santa Clara, CA, USA) hybridization oven. Slides were scanned using an Agilent G2565AA scanner and raw data were extracted using the QuantArray v3.0 software (PerkinElmer, Waltham, MA, USA).

### Preparation of yeast polysomal RNA

Polysomes were isolated as previously described by Arava [94], with minor modifications. For each sample, yeast cultures (80 mL) were harvested by centrifugation at 4,000 rpm, for four minutes, at 4°C, in the presence of 100 μg/mL cycloheximide to freeze protein synthesis elongation. Cells were then washed twice using 2 mL of lysis buffer (20 mM Tris-HCl at pH 8.0, 140 mM KCl, 1.5 mM MgCl_2_, 0.5 mM dithiothreitol, 100 μg/mL cycloheximide, 1 mg/mL heparin, 1% Triton X-100), and were resuspended in 700 μl of the same buffer supplemented with 0.6 volumes of chilled glass beads. Cell lysis was carried out using eight cycles of 30 seconds vortexing and 1 minute cooling on ice. Lysates were transferred to clean microfuge tubes and centrifuged for five minutes at 8,000 rpm at 4°C. Supernatants were transferred to clean microfuge tubes and 40 units A_280 nm _of sample were loaded onto 11 mL 15% to 50% sucrose gradients containing 20 mM Tris-HCl at pH 8.0, 140 mM KCl, 5 mM MgCl_2_, 0.5 mM dithiothreitol, 100 μg/mL cyclohexamide, 500 μg/ml heparin. Gradients were centrifuged at 35,000 rpm for 2 hours and 45 minutes, using a SW41 rotor and an Optima series ultracentrifuge (Beckman Coulter, Brea, CA, USA). Polysomal profiles were visualized by monitoring RNA absorbance at 254 nm using a Bio-Rad (Hercules, CA, USA) Biologic LP system adapted for this assay. The polysomal fraction of the gradient was recovered and RNA was precipitated as previously described by Arava [94]. mRNA was isolated from polysomal RNA using Oligotex (Qiagen, Hilden, Germany) beads and cDNA synthesis was carried out using 3 μg of purified mRNA. Labeling and hybridization were carried out as described above.

### Normalization and analysis of DNA microarray data

Raw data were normalized using *limmaGUI *software (R/Bioconductor, Boston, MA, USA) [95] and print-tip *lowess *normalization within arrays. Heatmaps and clustering of genes were carried out using MeV software [96]. Functional analysis of expression data obtained was carried out using the EXPANDER software (Algorithms in Computational Genomics group, Blavatnik School of Computer Science, Tel Aviv University, Tel Aviv, Israel) [97] and the YEASTRACT online tool (Biological Sciences Research Group, IBB and Knowledge Discovery and Bioinformatics group, INESC-ID, Lisbon, Portugal) [98] as well as the R/Bioconductor *limma *package (R/Bioconductor, Boston, MA, USA) [95]. The microarray raw data were submitted to the ArrayExpress database (EMBL-EBI, Hinxton, UK) and are available under the accession codes E-MTAB-153 and E-MTAB-166.

### Gene expression deregulation analysis

Differentially expressed genes (DEGs) for each of the mistranslations' time-points were extracted using a linear model analysis (R/Bioconductor package *limma *[95], considering as differentially expressed a variation equal or higher than 2X or 1X between each time-point and the initial time-point. Only genes with a significance level below a Benjamini-Hochberg corrected *P*-value of 10^-3 ^were considered as differentially expressed. The relaxed 1X DEGs were used in order to avoid a low number of genes in the GO analysis which could raise spurious enriched GO terms and distort the data analysis. The more strict 2X DEGs were used for other analysis, namely for ESR comparisons. GO term enrichment for DEGs listed at each time-point was carried out using the hypergeometric test developed by Falcon and Gentleman in *GOstats *[99], applied over each GO biological process, and then selecting GO terms enriched with a *P*-value lower than 10^-3^. The GO terms considered for mistranslation are either enriched in four or more time points or have a significance level below a *P*-value of 10^-9 ^for a single time point. This method provided approximately 40 GO terms that, after manually removing redundant and generic terms, resulted in a dozen terms (Table 1). The hierarchical clustering was carried out by constructing an expression matrix containing the stress profiles of genes annotated in the enriched GO terms. The values of this matrix were also averaged by time point and GO term and stress conditions were clustered again (Figures 2C, 5B, 6B). For the comparison of ESR vs. mistranslation, the ESR up- and down-regulated gene lists (*ESRup*, *ESRdown*) from Gasch *et al. *[42] were compared with the mistranslation time-point specific DEGs and with the combination of these DEGs lists into a mistranslations DEGs list.

### Real time quantitative PCR

Total RNA was extracted from yeast cells and genomic DNA contamination was removed using DNase I (Invitrogen), followed by phenol extraction. Total RNA quantity and quality were accessed using the Nanodrop 1000 and Agilent 2100 Bioanalyzer systems, respectively. Total RNA (40 μg) was reverse-transcribed to cDNA using Superscript II RT enzyme (Invitrogen) and oligo dT (12 to 18) primers, following the manufacturer's recommendations. First-strand cDNA templates were then used for PCR amplification of short (100 to 150 bp) gene fragments using appropriate primers. PCRs were carried out in triplicate using a Power SYBR Green PCR master mix (Applied Biosystems, Foster City, CA, USA) and analyzed using a 7500 real-time PCR system (Applied Biosystems), following the manufacturer's recommendations. A dissociation curve was generated at the end of each PCR cycle to check for primer dimerization. Standard dilution curves were determined for each primer set and their amplification efficiencies calculated. cDNA concentration in each sample was normalized to *ACT1*. Relative quantification of target cDNA was determined by calculating the difference in cross-threshold (Ct) values after normalization to the *ACT1 *signal, according to the Pfaffl's method [100] and the Excel-based program REST (Technical University of Munich, Munich, Germany) [101].

For RT-PCR, total RNA extracts were prepared as above from T0', T40', T90' and T180'. RNA samples were prepared for *HAC1 *mRNA for reverse transcription (see above) and RT-PCR using the PCR primers 5'-ATGACTGATTTTGAACTAACTAG and 5'-CAATTCAAATGAATTCAAACCTG.

### Protein pulse labeling with [^14^C]-Leucine

Amino acid incorporation was performed at time points T0', T40', T90', T180' and T240' after inducing the gene mistranslations with tetracycline. Briefly, 2 × 10^7^cells were collected and resuspended into 2 ml of pre-warmed minimal medium, 20 μl of cold [^14^C(U)]-L-Amino Acid Mixture were added, (Perkin Elmer, 0.1 mCi/ml) and the mixture was incubated 10 minutes at 30°C with agitation. Amino acid incorporation was stopped by the addition of 60 μl of cicloheximide (20 mg/ml) and ice incubation. Cells were washed once with cold water and frozen at -80°C. Protein was then extracted by resuspending cell pellets in 200 μl Lysis buffer (50 mM potassium phosphate buffer pH 7, 1 mM EDTA, 5% (vol/vol) glycerol, 1 mM phenylmethylsulfonyl fluoride, and complete mini protease inhibitor cocktail (Roche) and 120 μl of glass beads. Cells were disrupted using a Precellys (Bertin Technologies, Montigny-le-Bretonneux, France) disrupter (5 cycles of 10 sec at 5,000 rpm and 1 minute on ice between cycles) and centrifuged at 3,000 × g for 10 minutes. A total of 30 μl of supernatant was applied on 1 cm^2 ^square paper microfiber filter (GF/C, Whatman, Maidstone, UK). Amino acid incorporation was measured using a scintillation counter (Beckman) and protein extracts were quantified using the BCA protein quantification Kit (Pierce. Rockford, IL, USA). [^14^C(U)]-L-Amino acid incorporation was normalized against the total protein for each sample and compared to Control amino acid incorporation at each time point.

## Abbreviations

[^14^C]-Leu: leucine labelled with carbon 14; aaRS: aminoacyl-tRNA synthetase; CLIPS: chaperones linked to protein synthesis; DEGs: deregulated genes; ER: endoplasmic reticulum; ESR: environmental stress response; GO: gene ontology; HSPs: heat shock proteins; Leu: leucine; Ser: serine; tet: tetracycline; UPR: unfolded protein response; UPS: ubiquitin-proteasome system.

## Competing interests

The authors declare that they have no competing interests.

## Authors' contributions

JAP and MAAS conceived and designed the study. JAP carried out inducible tRNA yeast strains construction, β-galactosidase activity assays, RNA isolation and labeling, preparation and analysis of yeast polysomes and polysomal RNA. JAP and LC performed yeast strains growth, Real-time and RT-PCRs, DNA microarray experiments and also normalization and analysis of microarray data. RS and MK performed bioinformatics analysis of gene expression deregulation. JS, ARB and ACG carried out β-galactosidase western blots, aggregation assays and protein pulse labeling with [^14^C]-Leucine. JAP drafted the manuscript. GRM and MAAS supervised the study and corrected the manuscript. All the authors read and approved the final manuscript.

## Supplementary Material

Additional file 1 **Figure S1**. Growth curves of Control and *tetO*-tRNA clones when tRNA_CAG_^Ser ^is induced at OD_600 _= 0.1 (for further information see legend in Additional file 15). Click here for file

Additional file 2 **Figure S2**. Effect of mistranslation induction in yeast viability and re-grow in new medium (for further information see legend in Additional file 15). Click here for file

Additional file 3 **Figure S3**. Global yeast transcriptional responses to mRNA mistranslations and environmental stressors (for further information see legend in Additional file 15). Click here for file

Additional file 4 **Table S1**. DEGs found for each time point (for further information see legend below the table). Click here for file

Additional file 5 **Table S2**. Gene overlap between ESR and mistranslation DEGs for each time point (for further information see legend below the table). Click here for file

Additional file 6 **Figure S4**. Transcription profiles of the yeast core stress response (for further information see legend in Additional file 15). Click here for file

Additional file 7 **Table S3**. Series of tables representing gene expression response to mistranslation obtained in this study and all the others datasets discussed and compared along the publication. Click here for file

Additional file 8 **Figure S5**. Transcriptome profiles highlighting yeast chaperone and protein folding genes involved in the stress response (for further information see legend in Additional file 15). Click here for file

Additional file 9 **Figure S6**. Mistranslations and environmental stressors and their negative impact on the translational machinery (for further information see legend in Additional file 15). Click here for file

Additional file 10 **Figure S7**. Comparison of the translatome profiles of mistranslating cells at T90' with the translatome profiles of cells exposed to environmental stressors (for further information see legend in Additional file 15). Click here for file

Additional file 11 **Figure S8**. Mistranslation and environmental stressors and their impact in the unfolded protein response related genes (for further information see legend in Additional file 15). Click here for file

Additional file 12 **Figure S9**. Promoter elements that regulate the stress response induced by mistranslations (for further information see legend in Additional file 15). Click here for file

Additional file 13 **Figure S10**. Mistranslation and environmental stressors and their impact in the ubiquitin-proteasome pathway related genes (for further information see legend in Additional file 15). Click here for file

Additional file 14 **Figure S11**. Mistranslations affect stress and ribosome linked chaperone networks in a time dependent manner (for further information see legend in Additional file 15). Click here for file

Additional file 15 **Legends for all supplementary figures (Figures S1 to S11)**. Click here for file
