# Supplementary figures and images for: Low level genome mistranslations deregulate the transcriptome and translatome and generate proteotoxic stress in yeast
Source: BMC Biol. 2012 Jun 20;10:55. doi: 10.1186/1741-7007-10-55 (PMC3391182; doi:10.1186/1741-7007-10-55)

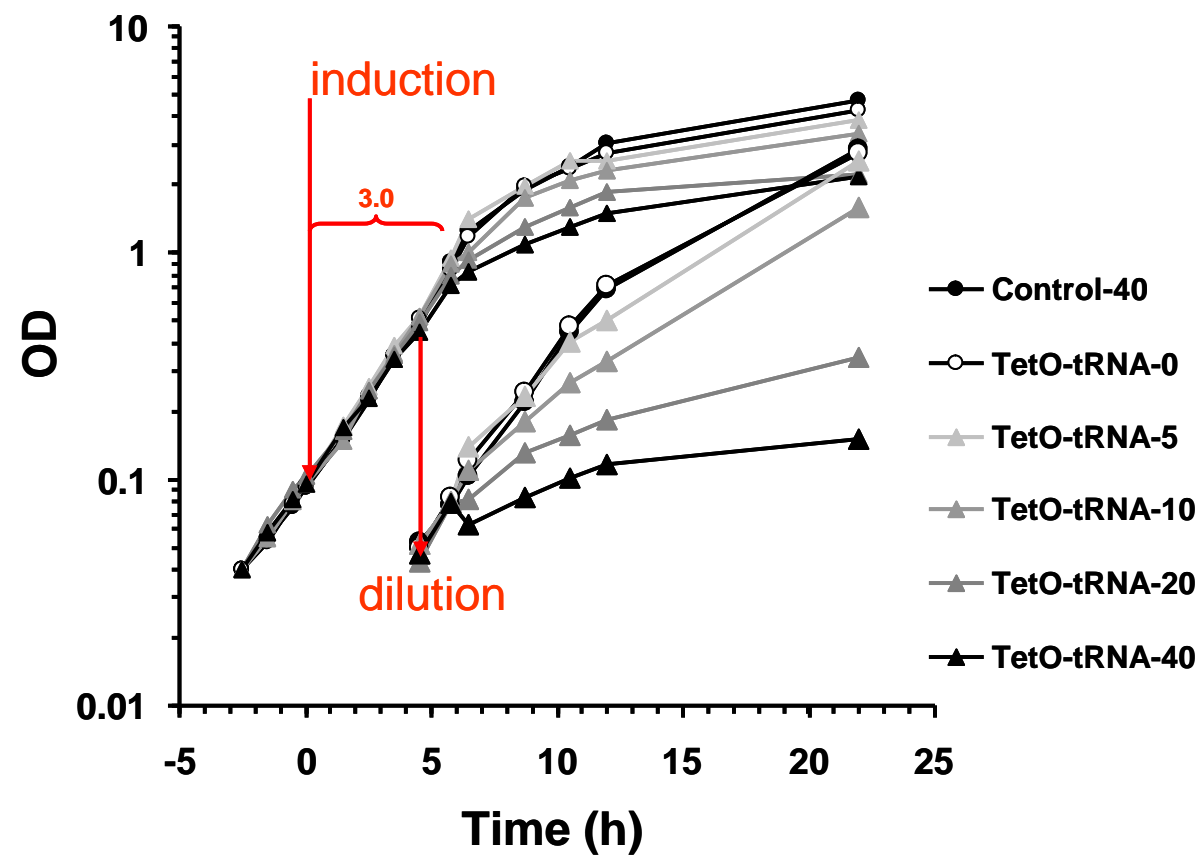

Figure S1

Supplement: Additional file 1 — Figure S1. Growth curves of Control and tetO-tRNA clones when tRNACAGSer is induced at OD600 = 0.1 (for further information see legend in Additional file 15). [file 1741-7007-10-55-S1.PDF]

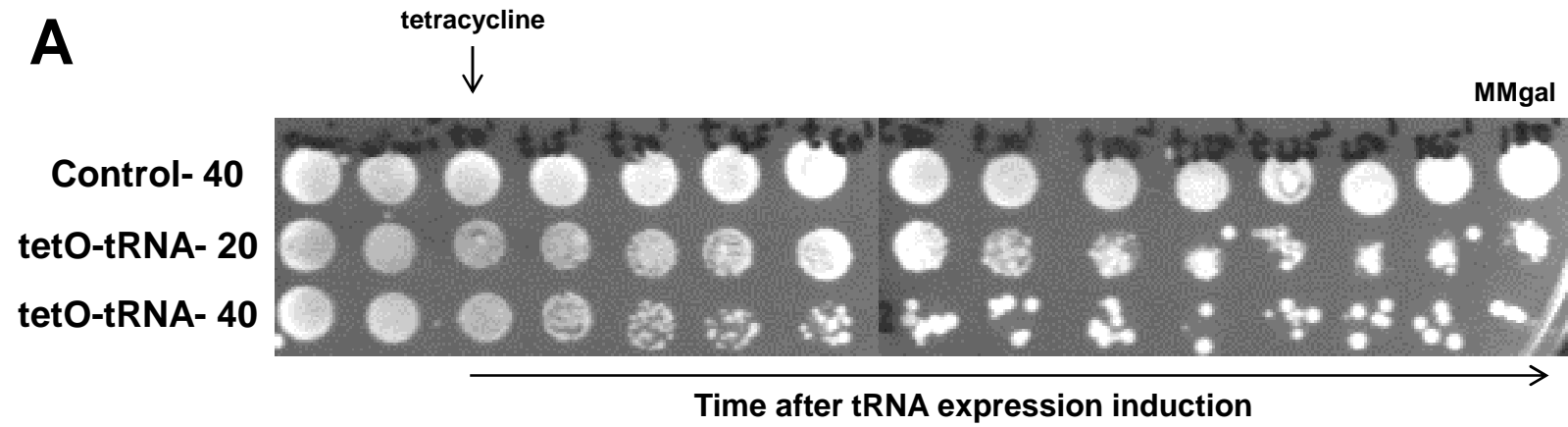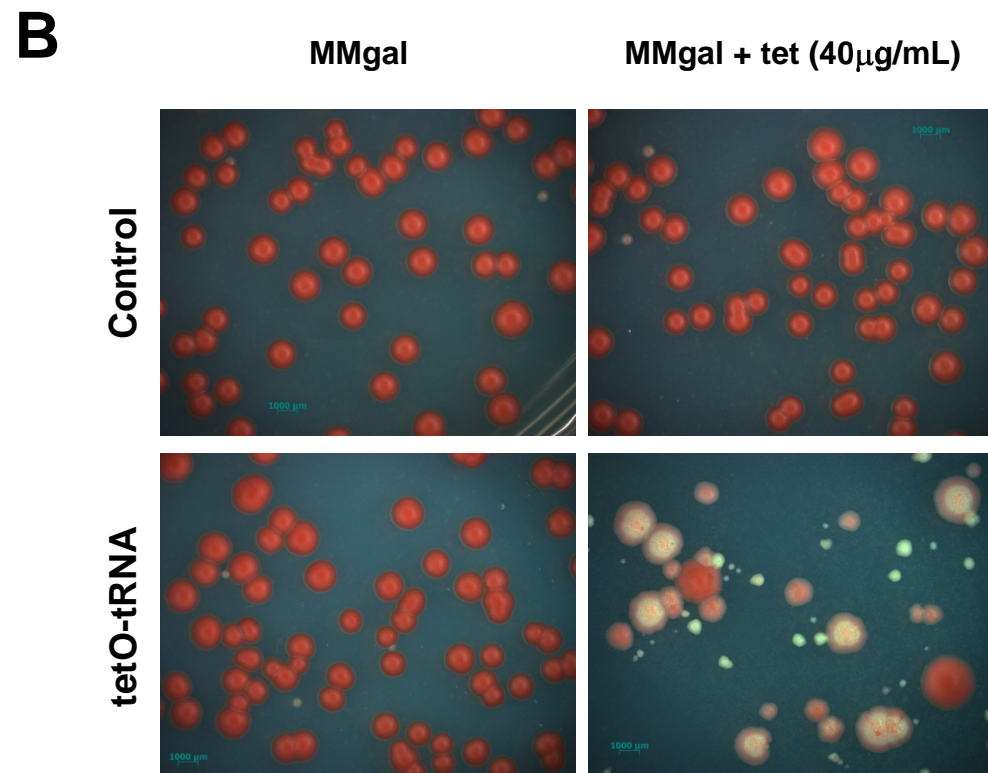

**Figure S2**

Supplement: Additional file 2 — Figure S2. Effect of mistranslation induction in yeast viability and re-grow in new medium (for further information see legend in Additional file 15). [file 1741-7007-10-55-S2.PDF]

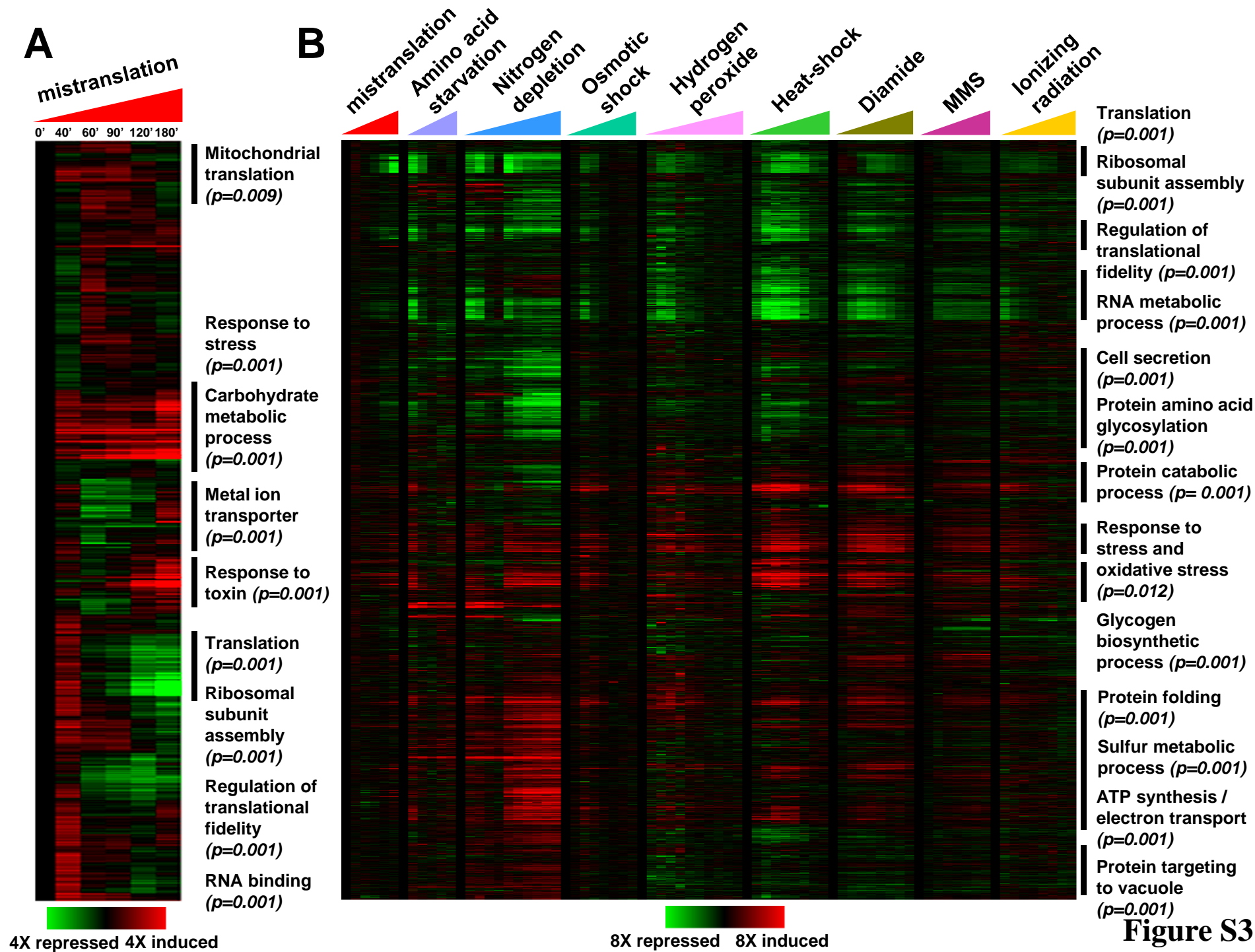

Supplement: Additional file 3 — Figure S3. Global yeast transcriptional responses to mRNA mistranslations and environmental stressors (for further information see legend in Additional file 15). [file 1741-7007-10-55-S3.PDF]

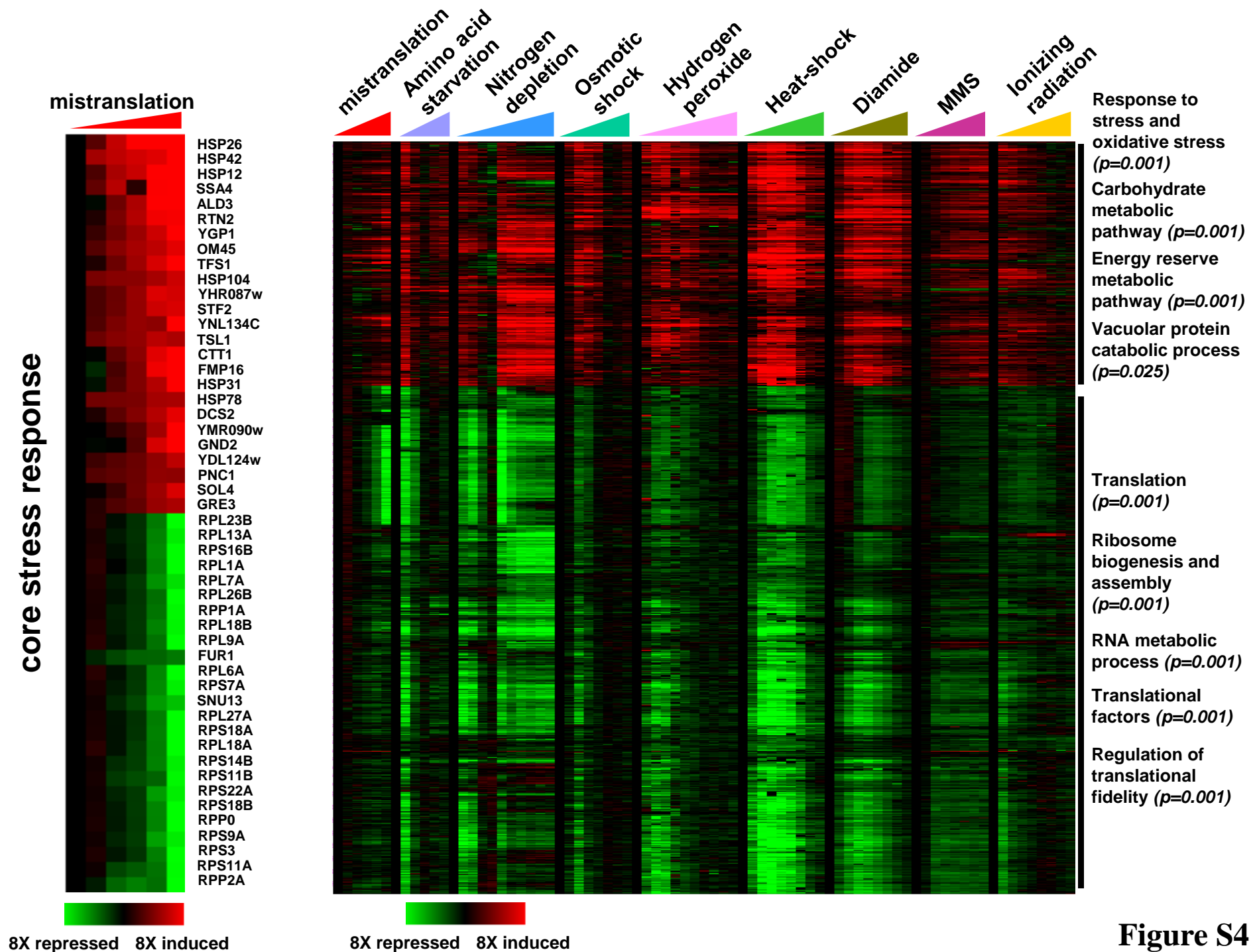

**Figure S4**

Supplement: Additional file 6 — Figure S4. Transcription profiles of the yeast core stress response (for further information see legend in Additional file 15). [file 1741-7007-10-55-S6.PDF]

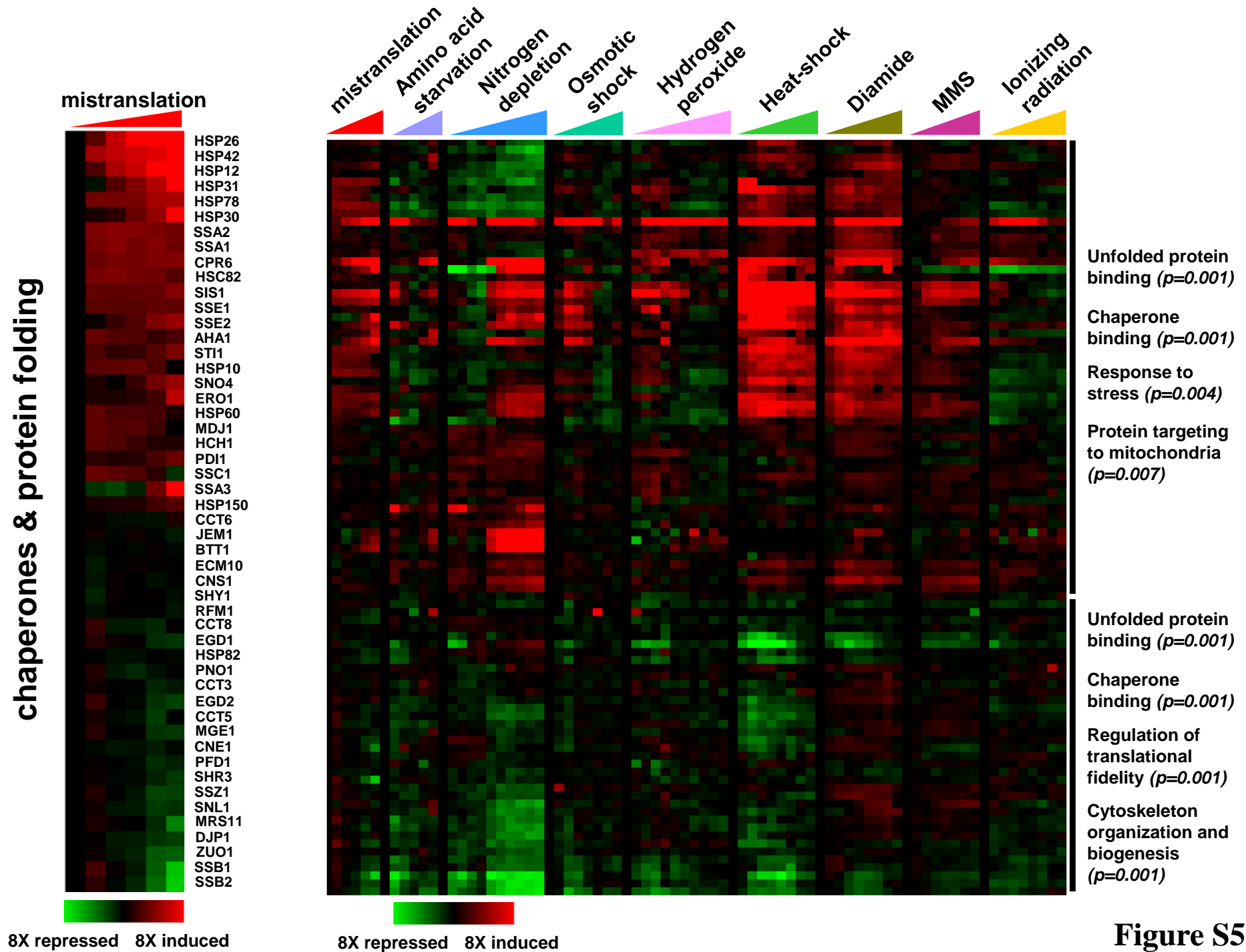

**Figure S5**

Supplement: Additional file 8 — Figure S5. Transcriptome profiles highlighting yeast chaperone and protein folding genes involved in the stress response (for further information see legend in Additional file 15). [file 1741-7007-10-55-S8.PDF]

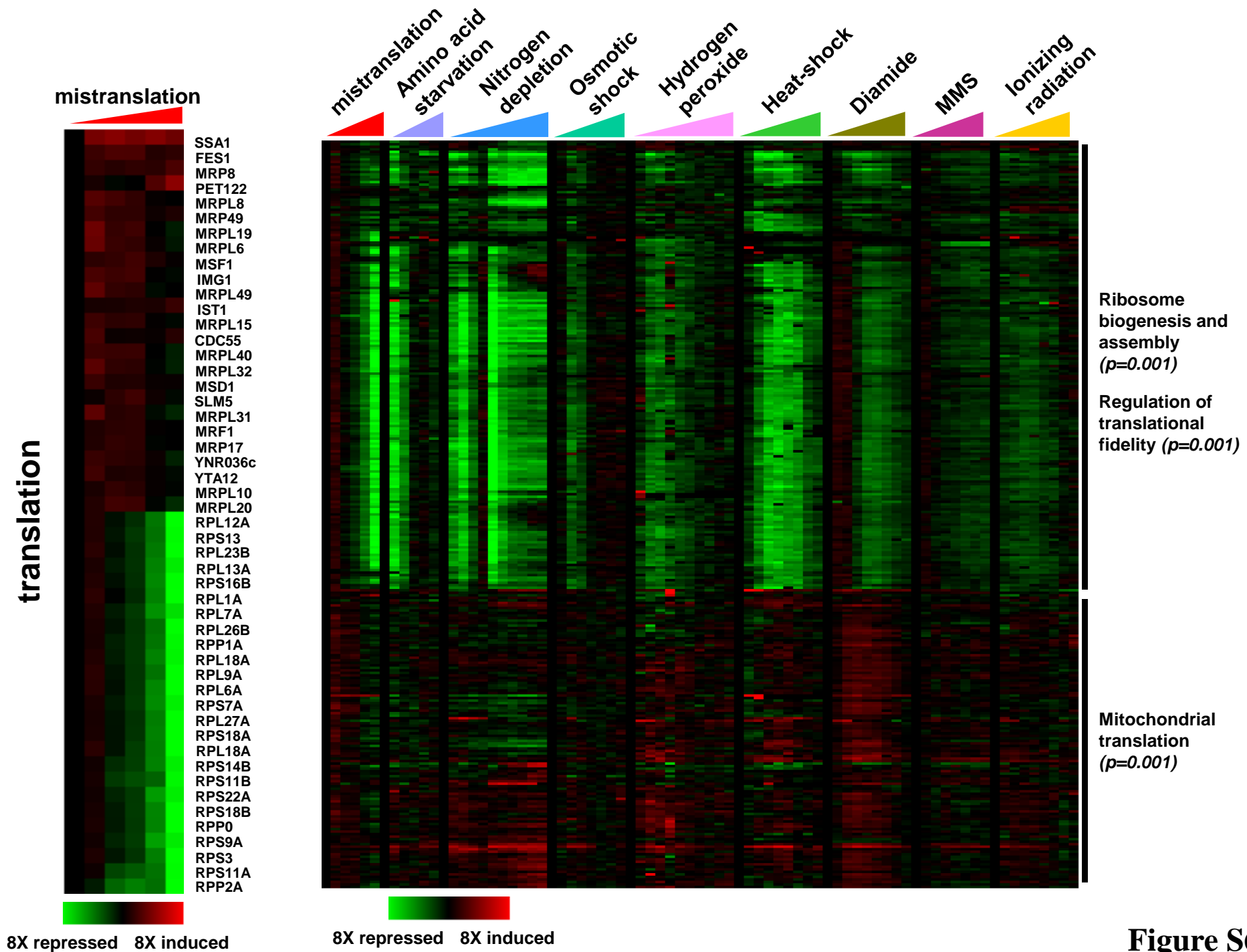

**Figure S6**

Supplement: Additional file 9 — Figure S6. Mistranslations and environmental stressors and their negative impact on the translational machinery (for further information see legend in Additional file 15). [file 1741-7007-10-55-S9.PDF]

# Translatome

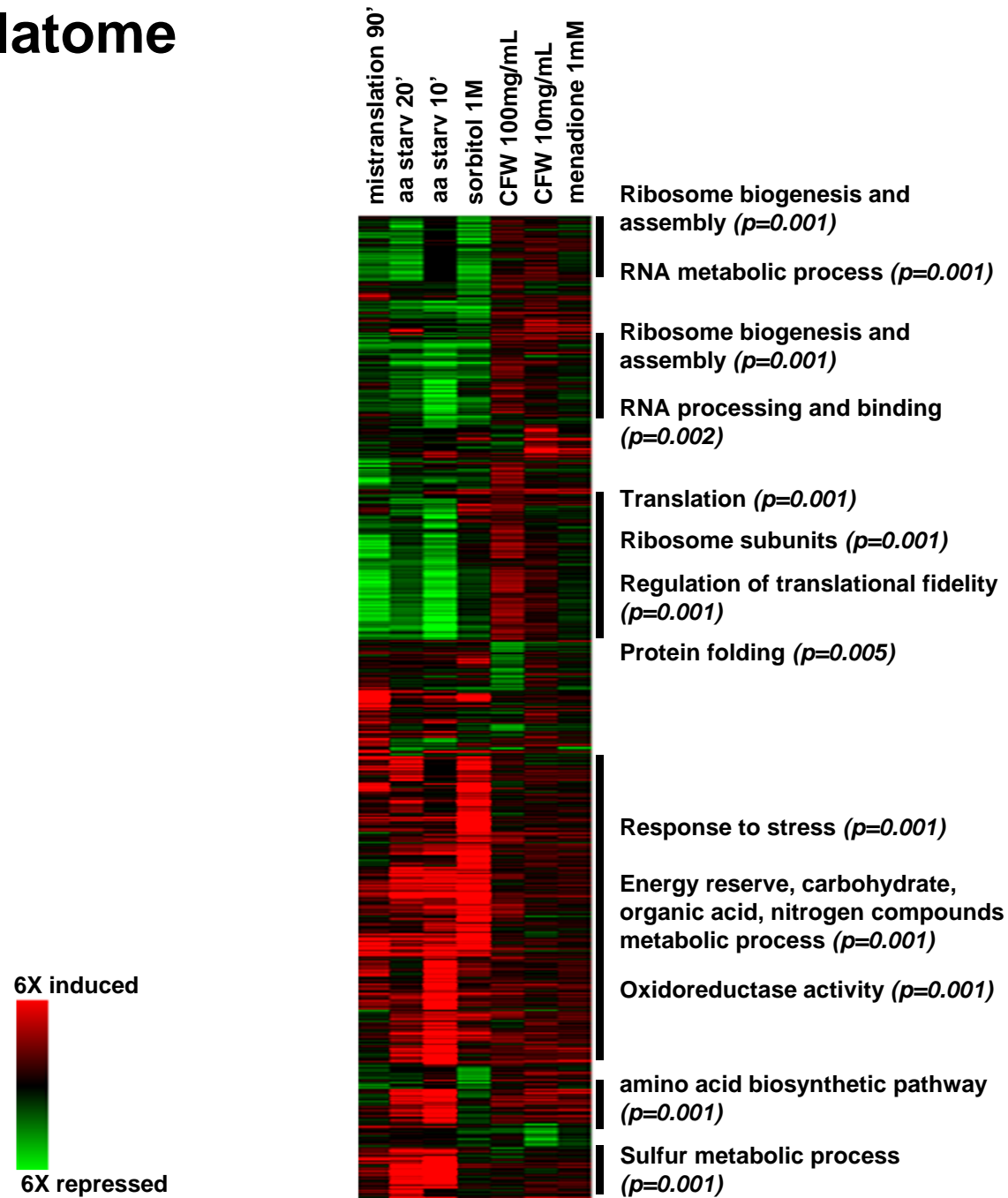

Figure S7

Supplement: Additional file 10 — Figure S7. Comparison of the translatome profiles of mistranslating cells at T90' with the translatome profiles of cells exposed to environmental stressors (for further information see legend in Additional file 15). [file 1741-7007-10-55-S10.PDF]

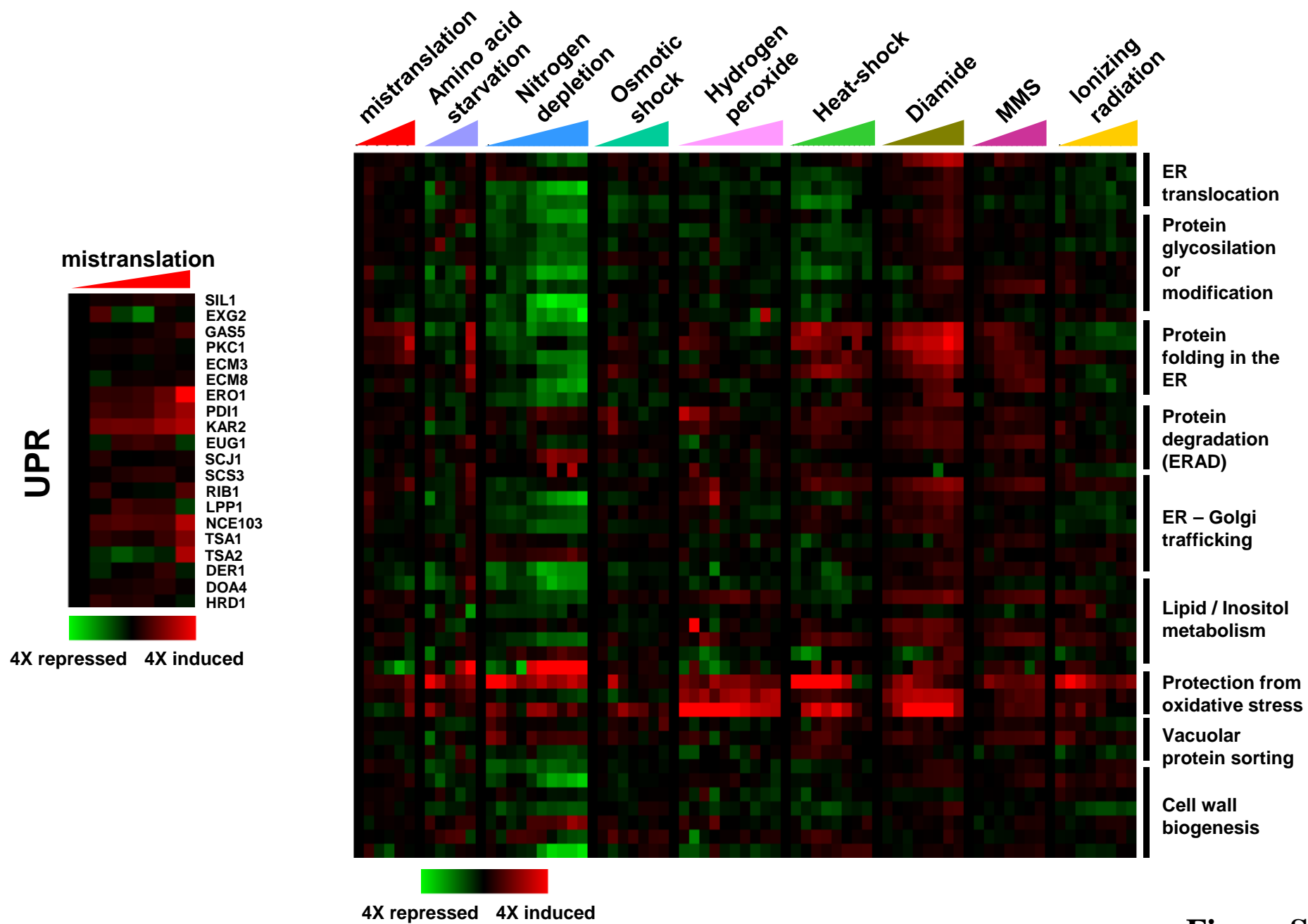

**Figure S8**

Supplement: Additional file 11 — Figure S8. Mistranslation and environmental stressors and their impact in the unfolded protein response related genes (for further information see legend in Additional file 15). [file 1741-7007-10-55-S11.PDF]

**A**

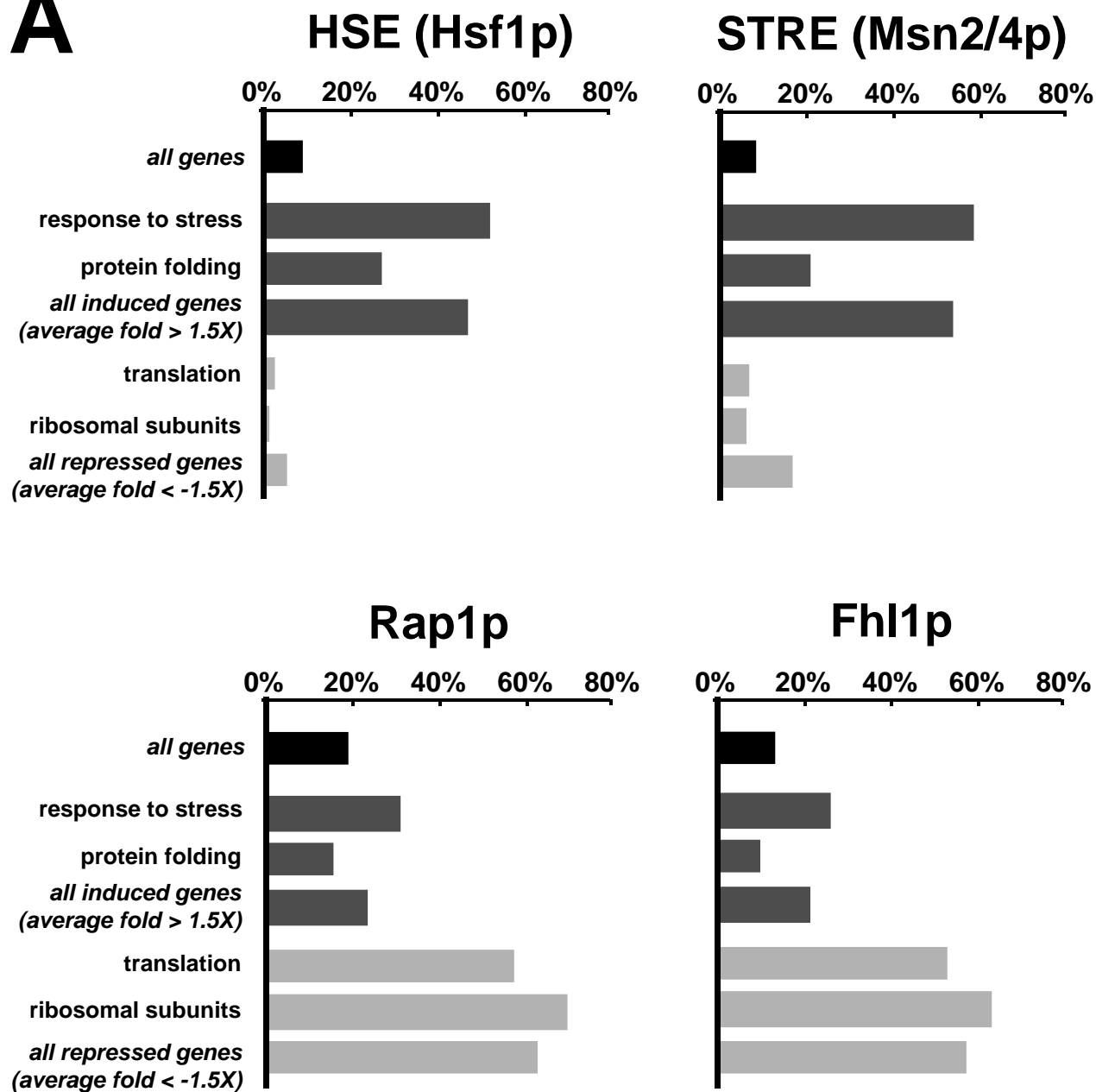

Stress Response regulation

Protein Synthesis regulation

**Figure S9**

**B**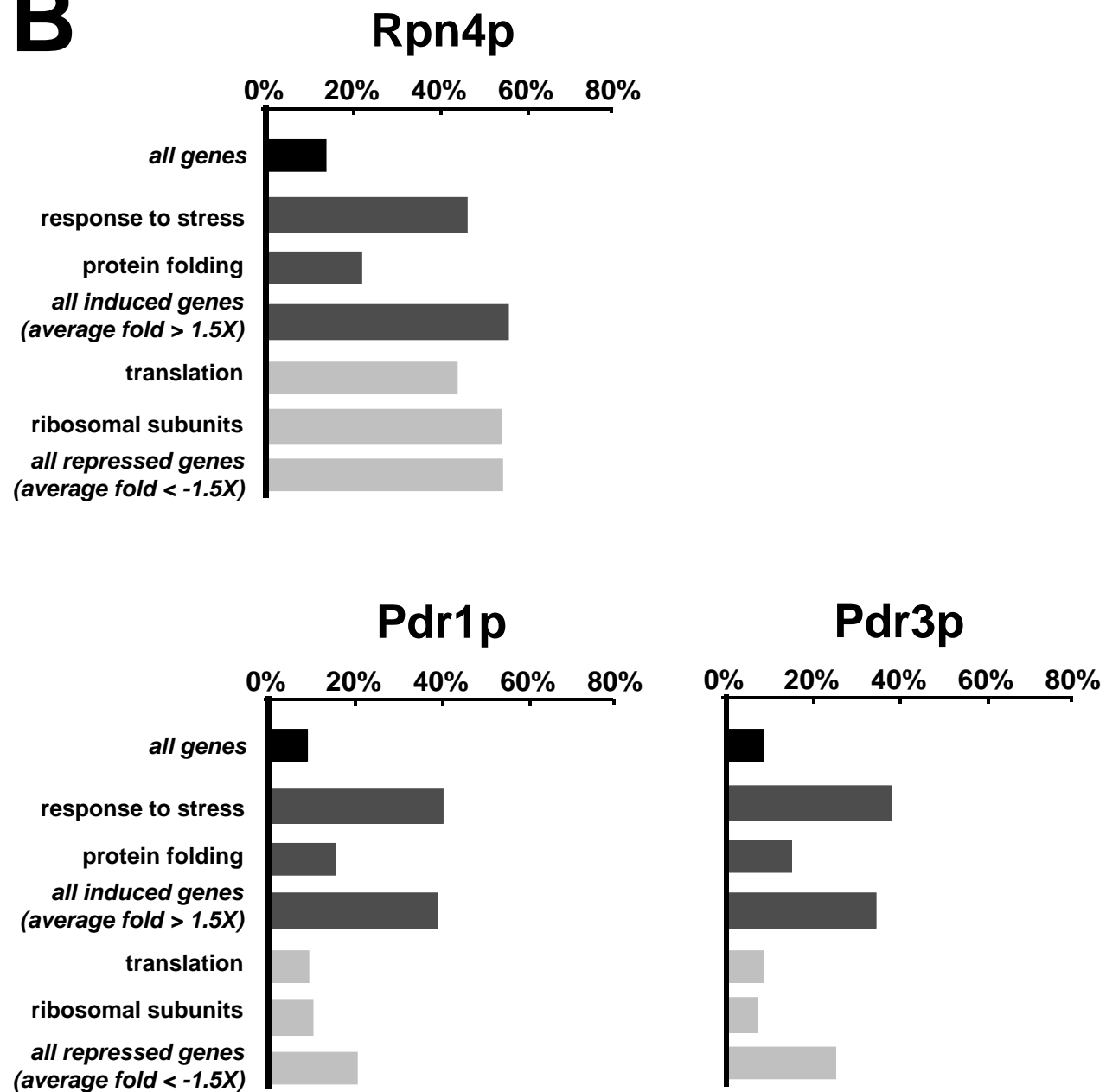**Figure S9 (cont.)**

Supplement: Additional file 12 — Figure S9. Promoter elements that regulate the stress response induced by mistranslations (for further information see legend in Additional file 15). [file 1741-7007-10-55-S12.PDF]

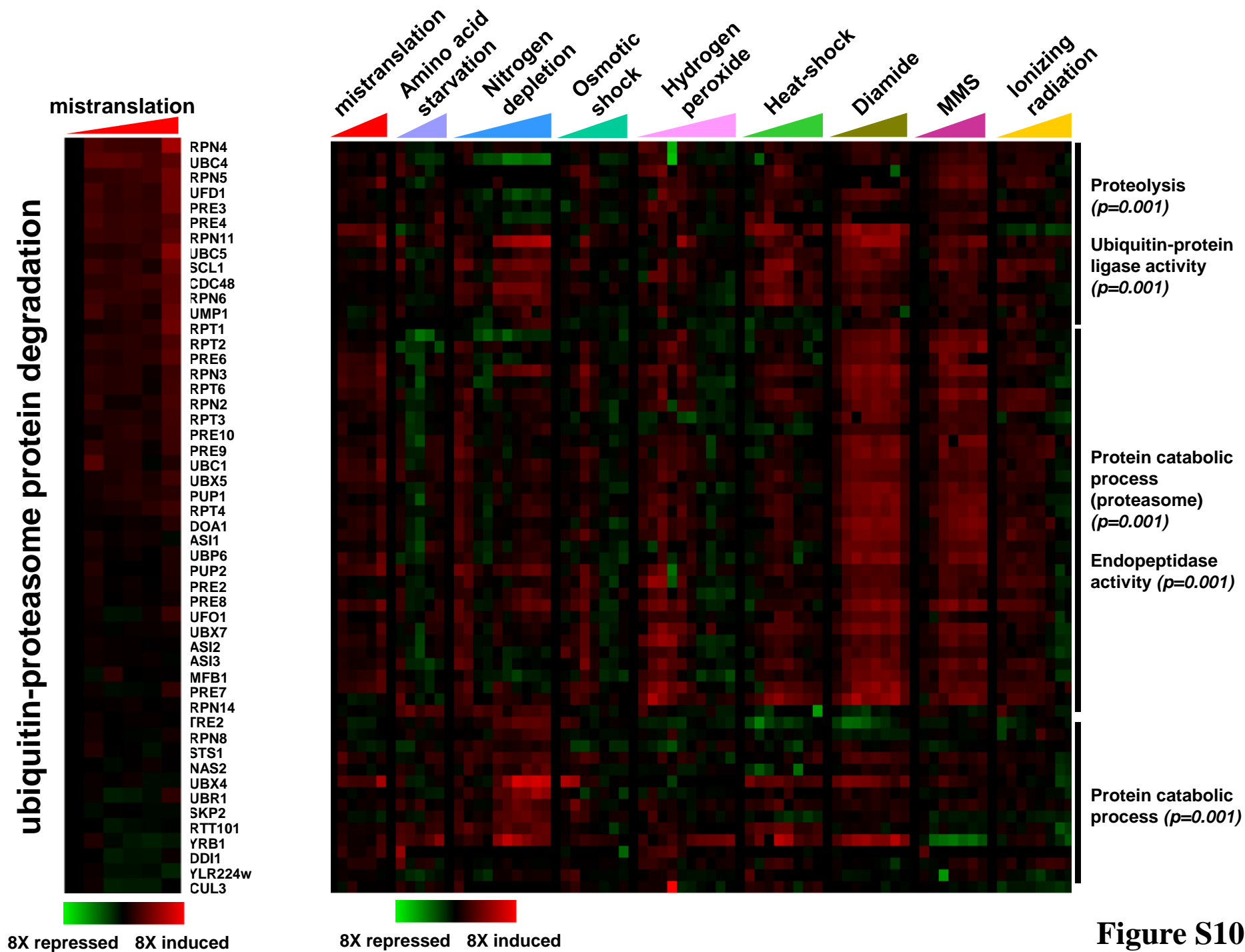

**Figure S10**

Supplement: Additional file 13 — Figure S10. Mistranslation and environmental stressors and their impact in the ubiquitin-proteasome pathway related genes (for further information see legend in Additional file 15). [file 1741-7007-10-55-S13.PDF]

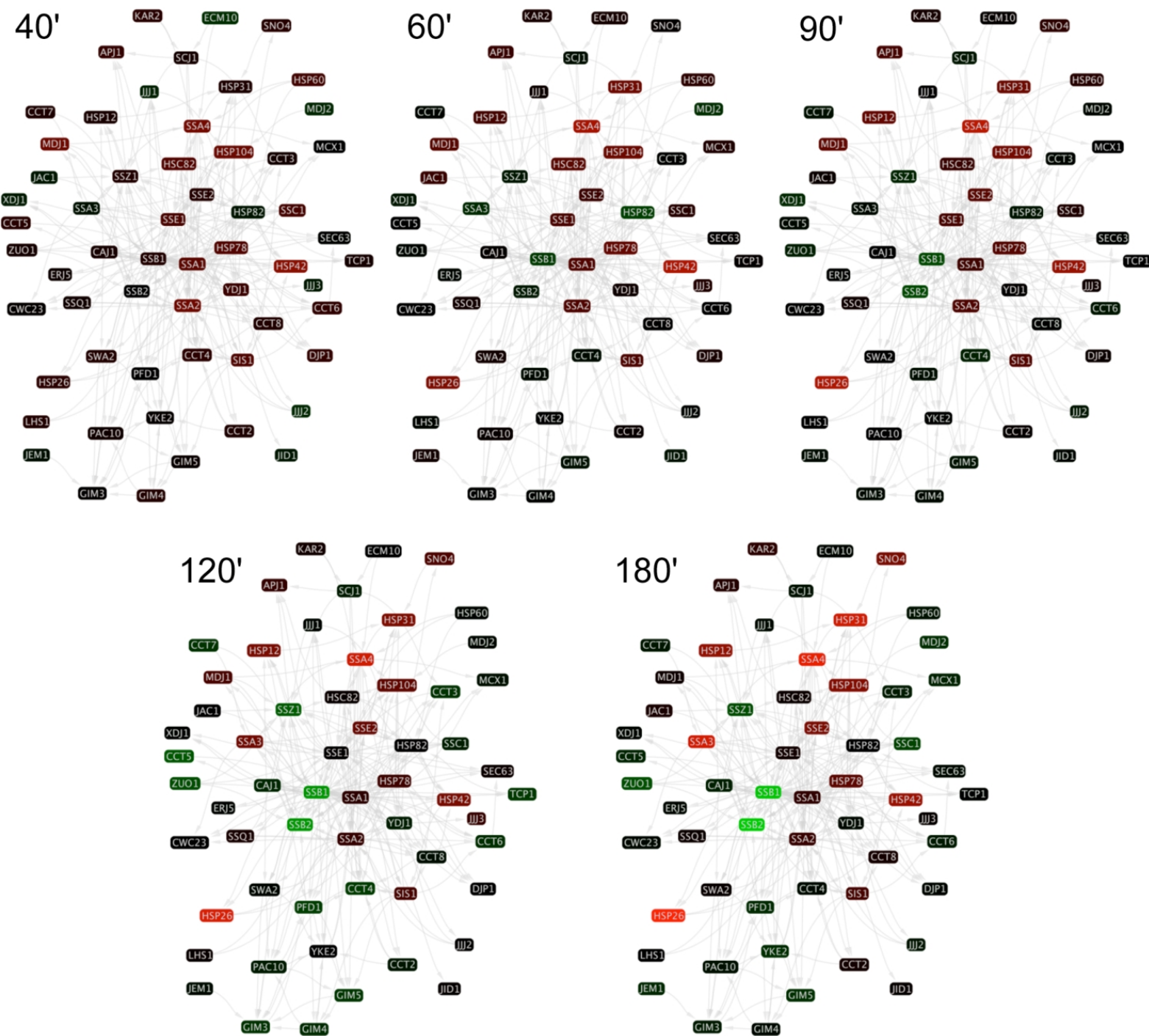

**Figure S11**

Supplement: Additional file 14 — Figure S11. Mistranslations affect stress and ribosome linked chaperone networks in a time dependent manner (for further information see legend in Additional file 15). [file 1741-7007-10-55-S14.PDF]
