# Supplementary material for: Low level genome mistranslations deregulate the transcriptome and translatome and generate proteotoxic stress in yeast
Source: BMC Biol. 2012 Jun 20;10:55. doi: 10.1186/1741-7007-10-55 (PMC3391182; doi:10.1186/1741-7007-10-55)
Supplement: Additional file 4 — Table S1. DEGs found for each time point (for further information see legend below the table). [file 1741-7007-10-55-S4.PDF]

Table S1. DEGs found for each time point.

DEGs for	# of genes	Gene list
0 min vs 40 min	12	***AAD6***, *BTN2, CUR1, HSP42, MBF1, MDJ1, MRPL31,* ***SSA4***, *TKL2,* ***TSL1***, *ZPR1*
0 min vs 60 min	25	*AAD10,* ***AAD6***, *BAG7, COB, COX2, FRM2, HAC1,* ***HSP26***, ***HSP31***, *MOH1, PHM8, PMA2, RRT1,* ***SSA4***, *TRX2,* ***TSL1***, *VAR1*
0 min vs 90 min	23	*AHP1, ALD3, CTT1, DCS2, FLR1, GPX2, HAC1, HSP12,* ***HSP26***, ***HSP31***, *MDJ1, OM45, PHM8,* ***SSA4***, *TFS1,* ***TSL1***, *YGP1*
0 min vs 120 min	33	***AAD6***, *AHP1, ALD3, CTT1, DAD3, DCS2, DDR2, FDH1, FDH2, FMP16, GLK1, GND2, HSP12,* ***HSP26***, ***HSP31***, *INO1, OM45, PAI3, PGM2, RPL20A, RPL23A, RPP0, RPS18A, SIP18, SOL4, SSA3,* ***SSA4***, *STF2, TFS1,* ***TSL1***, *YGP1, YRO2*
0 min vs 180 min	147	*AAD14, AAD16, AAD4,* ***AAD6***, *AHP1, ALD3, ASC1, ATG8, CTT1, DAD3, DCS2, DDR2, ECM4, FDH1, FDH2, FMP16, GND2, GRE1, GRE2, GTT2, HSP12,* ***HSP26***, *HSP30,* ***HSP31***, *HSP42, HXK2, ITR1, OM45, OYE3, PAI3, RPL11B, RPL12A, RPL12B, RPL13B, RPL14A, RPL14B, RPL16A, RPL16B, RPL17A, RPL17B, RPL18A, RPL18B, RPL19A, RPL19B, RPL1A, RPL1B, RPL20A, RPL21A, RPL22A, RPL23A, RPL23B, RPL24A, RPL24B, RPL25, RPL26A, RPL26B, RPL27A, RPL27B, RPL28, RPL29, RPL2A, RPL2B, RPL3, RPL30, RPL31A, RPL32, RPL33A, RPL34A, RPL34B, RPL35A, RPL35B, RPL36A, RPL37A, RPL37B, RPL38, RPL40A, RPL40B, RPL42A, RPL43A, RPL43B, RPL4A, RPL4B, RPL5, RPL6A, RPL7B, RPL8A, RPL8B, RPL9A, RPP0, RPP1A, RPP1B, RPP2A, RPP2B, RPS0A, RPS0B, RPS11A, RPS11B, RPS12, RPS13, RPS14A, RPS15, RPS16A, RPS17A, RPS17B, RPS18A, RPS18B, RPS19B, RPS1B, RPS2, RPS20, RPS21A, RPS21B, RPS22A, RPS22B, RPS23A, RPS24A, RPS24B, RPS26A, RPS27B, RPS28B, RPS3, RPS31, RPS4A, RPS4B, RPS6A, RPS6B, RPS7A, RPS8A, RPS8B, RPS9B, RTC3, RTN2, SIP18, SOD2, SSA3,* ***SSA4***, *SSB2, TFS1, TRX2,* ***TSL1***, *YGP1, YRO2*

TSL1 and SSA4 (in red and bold) are differentially expressed during the five time points studied. AAD6, HSP31, HSP26 (in bold) were differentially expressed during 4 time points. The higher number of differentially expressed genes at T180' is mainly due to the ribosomal protein genes (in blue, 93 genes). These genes were also found deregulated at the other time points if the differential expression threshold was lowered.
