# Supplementary material for: Low level genome mistranslations deregulate the transcriptome and translatome and generate proteotoxic stress in yeast
Source: BMC Biol. 2012 Jun 20;10:55. doi: 10.1186/1741-7007-10-55 (PMC3391182; doi:10.1186/1741-7007-10-55)
Supplement: Additional file 5 — Table S2. Gene overlap between ESR and mistranslation DEGs for each time point (for further information see legend below the table). [file 1741-7007-10-55-S5.PDF]

Table S2. Gene overlap between ESR and mistranslation DEGs for each time point.

DEG	#genes	% overlap (all), p-value	% overlap (up), p-value	% overlap (down), p-value
40 min	12	25%, $p>0.01$	25%, $p \approx 0.0156$	0%, $p > 0.01$
60 min	25	36%, $p \approx 0.0052$	36%, $p \approx 8.67\times10^{-7}$	0%, $p > 0.01$
90 min	23	57%, $p \approx 2.34\times10^{-6}$	57%, $p \approx 2.39\times10^{-12}$	0%, $p > 0.01$
120 min	33	70%, $p \approx 4.79\times10^{-13}$	58%, $p < 10^{-16}$	12%, $p > 0.01$
180 min	147	82%, $p < 10^{-16}$	16%, $p \approx 4.24\times10^{-8}$	66%, $p < 10^{-16}$

The second column indicates the total number of genes in each DEG list and the following columns contain their percentage in the ESR, ESRup and ESRdown gene lists, respectively.
