# Supplementary material for: Low level genome mistranslations deregulate the transcriptome and translatome and generate proteotoxic stress in yeast
Source: BMC Biol. 2012 Jun 20;10:55. doi: 10.1186/1741-7007-10-55 (PMC3391182; doi:10.1186/1741-7007-10-55)
Supplement: Additional file 15 — Legends for all supplementary figures (Figures S1 to S11). [file 1741-7007-10-55-S15.PDF]

SUPPLEMENTARY FIGURES

Figure S1. Growth curves of Control and *tetO*-tRNA clones growing in liquid MMgalactose+geneticin at 30ºC. Expression of the $tRNA_{CAG}^{Ser}$ was induced by adding several tetracycline concentrations at OD_{600nm} = 0.1 (T0´) and T90´. Yeast growth was monitored by measuring OD_{600} of the culture or by counting the number of cells per mL using a Neubauer cell counting chamber. Dilution indicates start of the second culture. The tetracycline concentrations tested are indicated in the inset (µg/mL). Number of generations is indicated in red.

Figure S2. Effect of mistranslation induction in yeast viability and re-grow in new medium. **A)** 5 µL of cultures from Control or *tetO*-tRNA yeast cells growing in liquid MMgalactose+geneticin at 30ºC were spotted in a MMgalactose agar plate at several time points before and after several concentrations of tetracycline were added to the culture. The first row (Control-40) corresponds to a culture of Control yeast cells in which 40 µg/mL were added at OD_{600nm}=0.4–0.5. The second and third rows (*tetO*-tRNA-20 and *tetO*-tRNA-40) correspond to cultures of *tetO*-tRNA cells, where 20 and 40 µg/mL were added at OD_{600nm}=0.4–0.5. The agar plates were incubated at 30ºC and after 4 days, pictures have been taken. **B)** Control and *tetO*-tRNA yeast cells were pre-culutred in MMgalactose+geneticin at 30ºC and then about 100-200 cells of each pre-culture have been spread in MMgalactose and MMgalactose+tetracycline agar plates. These plates were incubated at 30ºC during 4-5 days and then pictures have been taken.

Figure S3. Global yeast transcriptional responses to mRNA mistranslations and environmental stressors. **A)** Gene expression profiles of mistranslating cells obtained at

the time-points T0´, T40´, T60´, T90´, T120´ and T180´. **B)** Large scale comparison of mRNA profiles of yeast cells exposed to mistranslations (this study) and to various environmental stressors (data obtained from [1, 2]). Stress conditions are displayed in columns and genes in rows. Red indicates induction relative to T0´ and green indicates repression. Functional classes (GO terms) significantly enriched in the groups of genes highlighted in the heat maps were obtained using the TANGO software tool included in EXPANDER [3].

Figure S4. Transcriptome profiles of the yeast core stress response. The heat maps show a subset of stress response genes whose expression is deregulated by mistranslations (left panel) and environmental stressors (data from [1, 2]). The mistranslations time series shown represent the T0´-T180´ time points described in the manuscript. All stressors induce similar patterns of gene down- and up-regulation and this stress response signature involves down-regulation of translation components, namely translational factors, ribosome subunits protein, translational fidelity and genes involved in metabolic processes. Up-regulated genes are mainly involved in responses to oxidative stress (*CTT1*), carbohydrate and energy reserve metabolism (*GND2*, *SOL4* and *TSL1*) and protein folding, refolding and degradation (*HSP26*, *HSP42*, *HSP12*, *SSA4*, *HSP104*, *HSP31* and *HSP78*). The stress imposed by mistranslation has a similar effect on the core functional categories (GO terms) showing that mistranslated proteins are competent inducers of the stress response. GO terms significantly enriched in the groups of genes highlighted in the heat maps were obtained using the TANGO software tool of the EXPANDER software package [3].

Figure S5. Transcriptome profiles highlighting yeast chaperone and protein folding genes involved in the stress response. The heat maps show a subset of genes involved in protein folding whose expression is deregulated by mistranslations (left panel) and environmental stressors (data from [1, 2]). The mistranslations time series shown represents the T0´-T180´ time points. The heat maps show that mistranslations, heat-shock, diamide and MMS produce similar gene expression deregulation patterns which are characterized by up-regulation of genes involved in protein folding, like cytoplasmic chaperones and co-chaperones genes (*HSP26*, *HSP42*, *HSP104*, *SSA1*, *SSA2*, *HSC82*, *SIS1*, *SSE1*, *SSE2*, *AHA1*, *STI1* and *HCH1*), ER protein folding enzymes genes (*ERO1* and *PDI1*) and mitochondrial chaperones and protein targeting genes (*HSP78*, *HSP60*, *HSP10* and *SSC1*). Most of these genes are also deregulated by nitrogen starvation. The down regulation of molecular chaperones highlights chaperones associated to the translational machinery, the so called CLIPS, including ribosome-associated chaperones genes (*SSZ1*, *ZUO1*, *SSB1* and *SSB2*), nascent polypeptide-associated complex (NAC) genes (*EGD1*, *EGD2* and *CNS1*) and Cct-ring complex genes (*CCT3*, *CCT5*, *CCT6* and *CCT8*). The heat maps also show that stress affects cytoskeleton organization and structure and translation fidelity factors. Functional classes (GO terms) significantly enriched in the group of genes highlighted in the heat maps were obtained as described in Figure S3.

Figure S6. Mistranslations and environmental stressors have strong negative impact on the translational machinery. The heat maps show a subset of genes involved in protein synthesis whose expression is deregulated by mistranslation (left panel) and environmental stressors (data from [1, 2]). The mistranslations time series represent the T0´-T180´ time points described in the main text. The heat map shows that increased

mistranslations level have a strong negative impact on the translational machinery as most ribosomal protein genes, genes involved in ribosome biogenesis and mRNA translation fidelity, are strongly repressed (*RP* and several other genes). The heat maps produced by the environmental stressors show that amino acid starvation induces an initial repression of translational factors but cells recover over time likely due to induction of amino acid biosynthesis through activation of the Gcn4p transcriptional regulator. Nitrogen depletion and heat-shock have very strong negative impacts on the translational machinery, however the other stressors produce weaker effects. These gene expression profiles support the hypothesis that yeast cells sense and integrate the stress response induced by mistranslation as strong rather than weak stress (see main text). Interestingly, both mistranslations and environmental stressors deregulate mitochondrial translation, suggesting that mistranslated proteins also affect respiration and ATP synthesis. The mistranslation profile (right panel) shows weak up-regulation of mitochondrial translational factors (*MRP* and other genes) between T20´ and T90´ and slight down-regulation between T120´and T180´, suggesting that gradual increase in mistranslated proteins, likely in the mitochondrial matrix, down-regulates mitochondrial function. The pattern of expression of down-regulated genes shows stress specificity which is not observed for the general pattern of up-regulated genes.

Figure S7. The translatome of mistranslating cells. Comparison of the translatome profiles of mistranslating cells at T90´ with the translatome profiles of cells exposed to the environmental stressors indicated. The translatome data of cells exposed to environmental stresses was obtained from Halbeisen et al. [4] Stress conditions are displayed in columns and genes in rows. Red indicates gene up-regulation relative to T0´ and green indicates repression. Functional classes (GO terms) significantly enriched

in the groups of genes highlighted in the heat maps were obtained using the TANGO software tool of EXPANDER [3].

Figure S8. Mistranslations and environmental stressors deregulate the unfolded protein response. Mistranslations had a mild effect on the expression of UPR genes, however *ERO1*, *PDI1*, *KAR2* (protein folding), *TSA1* and *TSA2* (response to oxidative stress) were up-regulated, indicating that mistranslations increased the level of misfolded proteins in the ER. Comparison of the UPR heat maps produced by mistranslations and environmental stressors show that each stressor produces a specific UPR signature and that heat-shock, diamide, MMS have stronger effect on up-regulation of genes involved in protein folding and degradation (ERAD – *DER1*, *DOA4* and *HRD1*) and also in the protection against oxidative stress (*NCE103*, *TSA1* and *TSA2*). The maps also suggest that ER stress down-regulates genes involved in protein translocation (*SIL1*) with some exceptions, like diamide and mistranslations. Highlighted functional classes in the heat map were based in those described previously by Travers et al. [5].

Figure S9. Promoter elements that regulate the stress response induced by mistranslations. **A)** Promoter analysis of cis-regulatory elements showed that the yeast transcription factors (TF) Hsf1p, Msn2p/4p, Rap1p and Fhl1p were the main regulators of the stress response induced by mistranslations. For this analysis, all genes in the yeast genome, all genes up-regulated by mistranslations, all genes down-regulated by mistranslations and genes involved in the response to stress, protein folding, translation process and those encoding ribosomal subunits, were considered in the promoter analysis. The promoter enrichment analysis was carried out using the YEASTRACT online tool [6]. **B)** A similar promoter enrichment analysis for the cis-elements

recognized by Rpn4p, Pdr1p and Pdr3p which regulate transcription of proteasome and drug resistance related genes. For each TF analysis all yeast genes, all genes up-regulated by mistranslations, all genes down-regulated by mistranslations and the genes involved in response to stress only, protein folding, translation process and those encoding ribosomal subunits, were considered in this analysis.

Figure S10. Mistranslations and environmental stressors deregulate the ubiquitin-proteasome pathway. The heat maps show a subset of genes involved in the ubiquitin-proteasome pathway whose expression is deregulated by mistranslation (left panel) and environmental stressors (data from [1, 2]). The mistranslations time series shown represent the T0´-T180´ time points described in the main manuscript. The heat maps show that the expression pattern of genes deregulated by mistranslations is similar to those of environmental stressors and involves up-regulation of ubiquitin-protein ligase (*UBC1*, *UBC4*, *UBC5* and *ASI1*), proteasome subunits (*RPN4*, *RPN5*, *RPN11*, *RPT1*, *RPT2*, *PRE3* and *PRE4*, among other genes) and endopeptidase activity (*SCL1* and *PUP1*) related genes. However, the average gene fold variation induced by mistranslations and environmental stressors is lower for the ubiquitin-proteasome genes than for the other stress related gene sub-categories. Functional classes (GO terms) significantly enriched in the group of genes highlighted in the heat maps were obtained as described in Figure S3.

Figure S11. Mistranslations affect stress and ribosome linked chaperone networks in a time dependent manner. The effects of mistranslations on the expression of stress (HSP) and ribosome linked (CLIPS) chaperone networks was analyzed along the mistranslations time series studied (T0´to T180´). For this, we have used the chaperone

network available at the ChaperoneDB [7], visualized it using the BicOverlapper software [8], using a force directed layout with loose edge lengths in order to clarify the highly connected network. Nodes were coloured with the normalized expression levels of the transcriptional profiling, for every time point. The overall expression level of most chaperone genes was mild from T40´ to T90´, with slight up regulation of *SSA1*, *SSA2*, *SSA4* (chaperone *HSP70*) and small chaperones *HSP42* and *HSP26*. At T90´ the highly interconnected *SSB1* and *SSB2* chaperones of the *HSP70* family were strongly down regulated. *SSA3* and *SSA4* were also highly up regulated after T90´ along with the small chaperones *HSP26* and *HSP31*. The expression of *HSP90* genes (*HSP82*, *HSC82*, at the center-top and center right) was low from T40´ to T90´ and returned back to normal at T120´. The maps show the high interconnectivity of the yeast chaperones and highlight the impact of mistranslations on their networks.

References

1. Gasch AP, Huang M, Metzner S, Botstein D, Elledge SJ, Brown PO: **Genomic expression responses to DNA-damaging agents and the regulatory role of the yeast ATR homolog Mec1p**. *Mol Biol Cell* 2001, **12**(10):2987-3003.
2. Gasch AP, Spellman PT, Kao CM, Carmel-Harel O, Eisen MB, Storz G, Botstein D, Brown PO: **Genomic expression programs in the response of yeast cells to environmental changes**. *Mol Biol Cell* 2000, **11**(12):4241-4257.
3. Shamir R, Maron-Katz A, Tanay A, Linhart C, Steinfeld I, Sharan R, Shiloh Y, Elkon R: **EXPANDER--an integrative program suite for microarray data analysis**. *BMC Bioinformatics* 2005, **6**:232.
4. Halbeisen RE, Gerber AP: **Stress-Dependent Coordination of Transcriptome and Translatome in Yeast**. *PLoS Biol* 2009, **7**(5):e105.
5. Travers KJ, Patil CK, Wodicka L, Lockhart DJ, Weissman JS, Walter P: **Functional and genomic analyses reveal an essential coordination between the unfolded protein response and ER-associated degradation**. *Cell* 2000, **101**(3):249-258.
6. Teixeira MC, Monteiro P, Jain P, Tenreiro S, Fernandes AR, Mira NP, Alenquer M, Freitas AT, Oliveira AL, Sa-Correia I: **The YEASTRACT database: a tool for the analysis of transcription regulatory associations in Saccharomyces cerevisiae**. *Nucleic Acids Res* 2006, **34**(Database issue):D446-451.
7. Gong Y, Kakihara Y, Krogan N, Greenblatt J, Emili A, Zhang Z, Houry WA: **An atlas of chaperone-protein interactions in Saccharomyces cerevisiae:**

implications to protein folding pathways in the cell. *Mol Syst Biol* 2009, **5**:275.
8. Santamaria R, Theron R, Quintales L: **BicOverlapper: a tool for bicluster visualization**. *Bioinformatics* 2008, **24**(9):1212-1213.
